# Delivering Antisense Oligonucleotides across the Blood‐Brain Barrier by Tumor Cell‐Derived Small Apoptotic Bodies

**DOI:** 10.1002/advs.202004929

**Published:** 2021-05-04

**Authors:** Yulian Wang, Jiayun Pang, Qingyun Wang, Luocheng Yan, Lintao Wang, Zhen Xing, Chunming Wang, Junfeng Zhang, Lei Dong

**Affiliations:** ^1^ State Key Laboratory of Pharmaceutical Biotechnology School of Life Sciences Nanjing University 163 Xianlin Avenue Nanjing 210093 China; ^2^ State Key Laboratory of Quality Research in Chinese Medicine Institute of Chinese Medical Sciences University of Macau Taipa Macau SAR 999078 China; ^3^ Chemistry and Biomedicine Innovation Center Nanjing University Nanjing Jiangsu 210023 China

**Keywords:** blood‐brain barrier, CD44v6, small apoptotic bodies, transcytosis, tumor cells

## Abstract

The blood‐brain barrier (BBB) is the most restrictive and complicated barrier that keeps most biomolecules and drugs from the brain. An efficient brain delivery strategy is urgently needed for the treatment of brain diseases. Based on the studies of brain‐targeting extracellular vesicles (EVs), the potential of using small apoptotic bodies (sABs) from brain metastatic cancer cells for brain‐targeting drug delivery is explored. It is found that anti‐TNF‐*α* antisense oligonucleotide (ASO) combined with cationic konjac glucomannan (cKGM) can be successfully loaded into sABs via a transfection/apoptosis induction process and that the sABs generated by B16F10 cells have an extraordinarily high brain delivery efficiency. Further studies suggest that ASO‐loaded sABs (sCABs) are transcytosed by b. End3 (brain microvascular endothelial cells, BMECs) to penetrate the BBB, which is mediated by CD44v6, and eventually taken up by microglial cells in the brain. In a Parkinson's disease (PD) mouse model, sCABs dramatically ameliorate PD symptoms via the anti‐inflammatory effect of ASO. This study suggests that sABs from brain metastatic cancer cells are excellent carriers for brain‐targeted delivery, as they have not only an extraordinary delivery efficiency but also a much higher scale‐up production potential than other EVs.

## Graphical abstract

Cancer cells that spread to the brain naturally produce nano‐sized “apoptotic bodies” (ABs), which can package nucleic acid drugs efficiently (>21.3%), cross the brain‐blood barrier via transcytosis mediated by CD44v6 exposed on ABs, and deliver anti‐inflammatory nucleotides into microglial cells. These ABs show potential as carriers for the treatment and diagnosis of brain diseases.

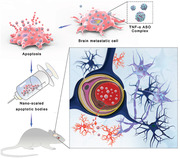

## 1 Introduction

The delivery of drugs, especially macromolecule drugs, into the brain with sufficient efficiency is a major challenge for the treatment of brain diseases. In the past several decades, many studies on this subject have focused on the blood‐brain barrier (BBB), and various means have been developed to break the restriction of these physiological barriers with limited success in the laboratory. Recent studies have suggested that the mechanism of controlling the entry of materials into the brain is much more complex than we previously thought, and how the BBB or other related structures prevent the inflow of matter from circulation remains obscure.^[^ ^1^, ^2^, ^3^ ^]^ Without mechanism‐based guidance, strategies exploiting the natural route across the BBB should be taken into consideration.

Some types of cancer and immune cells are able to effectively migrate into the brain.^[^ ^4^, ^5^, ^6^, ^7^, ^8^ ^]^ Although the details of brain metastasis and brain infiltration are not yet completely understood, the capability of these cells to penetrate the BBB could be harnessed in some proper way. For instance, some studies have used extracellular vesicles (EVs) such as exosomes for brain‐targeted delivery,^[^ ^9^, ^10^, ^11^ ^]^ and another recent study demonstrated that exosomes engineered with surface‐integrated BBB‐targeting molecules were effective in mediating intrabrain drug delivery.^[^ ^12^, ^13^, ^14^, ^15^ ^]^ However, the uncontrollable drug‐loading process and the difficulty in scaling up the biogenesis of exosomes make exosomes and shedding vesicles far from adequate in regards to meeting pharmaceutical requirements.

Compared to exosomes produced by living cells, apoptotic cells can generate membrane‐enclosed apoptotic bodies (ABs) with much higher efficiency.^[^ ^16^, ^17^ ^]^ More importantly, the apoptotic process can be totally controlled with a standardized operation. Moreover, as apoptosis is a natural process in which a cell actively packages its biomolecules into vesicles, it can be used to load drugs, such as nucleic acids, into ABs with much higher efficiency than other EVs.^[^ ^16^, ^17^, ^18^, ^19^ ^]^ Additionally, the AB membrane is that of the native cell, and cytomembrane‐integrated protein molecules should therefore be preserved in a greater abundance than exosomes that are assembled within the cytoplasm, which is important when a special membrane protein is needed to mediate brain delivery.^[^ ^16^, ^17^, ^20^ ^]^

In the present study, we designed an AB‐based delivery system and tested its efficacy in delivering antisense oligonucleotide (ASO) into the brain to inhibit brain inflammation in a mouse Parkinson's disease (PD) model. After screening some cells with brain metastasis, we obtained drug‐loaded small ABs (sABs) from a melanoma cell line with high brain drug delivery capacity and tested their pharmaceutical properties and capability to treat PD. Furthermore, we identified the protein molecule CD44v6 as a mediator of the transportation process across the BBB.

## 2 Results

### 2.1 Preparation of Small Apoptotic Bodies Loaded with ASO

**Figure 1:**
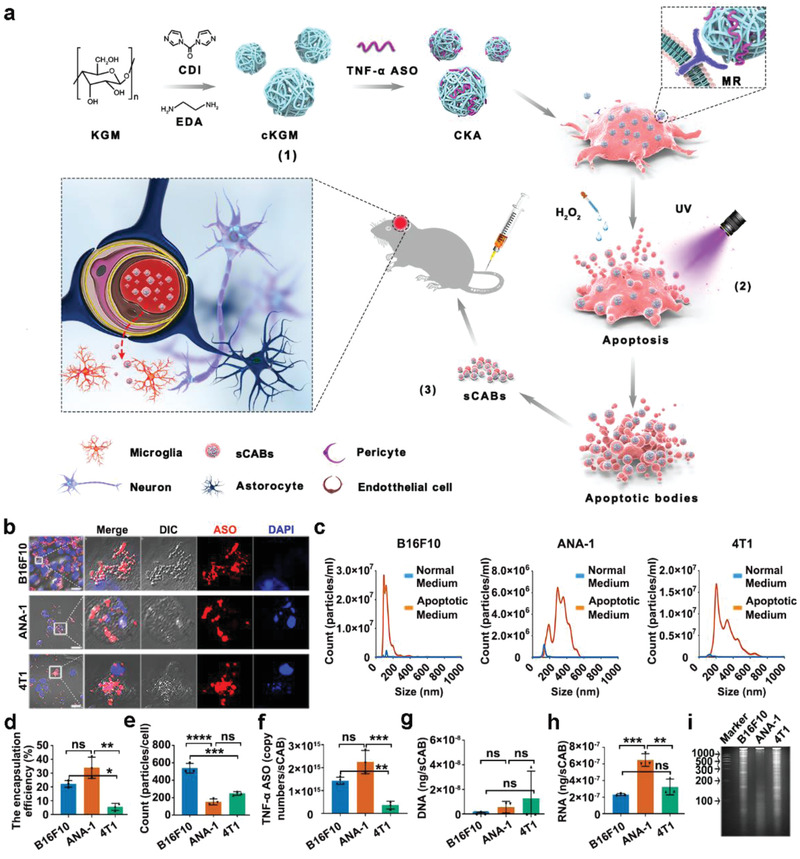
Preparation of sCABs. a) Schematic diagram of the protocol for producing sCABs and delivering ASO into the brain. b) Representative fluorescence microscopy images of sCABs generation from apoptotic cells containing Cy5‐ASO. Scale bars: 25 µm. c) The diameter distributions of different sCABs generated by B16F10, ANA‐1, and 4T1 cells and collected at 36 h after apoptosis. d) The encapsulation efficiencies of sCABs for ASO calculated as the ratio of sCABs‐encapsulated ASO to ASO added into the cell culture. e) The numbers of sCABs secreted from a single cell. f) The average ASO copy numbers in a single sCAB. g,h) The concentrations of DNA (g) and RNA (h) in sCABs. i) Electrophoretic analysis of RNA purified from different sCABs. Three sCABs analyzed in this section were collected at 36 h after apoptosis. Images are representative of three independent experiments. Representative results are presented as the means ± SD; *n* = 3 per group. *p*‐values are calculated using one‐way ANOVA followed by Bonferroni's multiple comparisons post hoc test (Figure 1d–h), ^*^ *p* < 0.05, ^**^ *p* < 0.01, ^***^ *p* < 0.001, and ^****^ *p* < 0.0001.

In our study, we chose three cell lines reported to have high brain metastasis capacity, 4T1 (mouse breast cancer cells), B16F10 (mouse melanoma cells), and ANA‐1 (immortalized mouse peritoneal macrophages), to generate ABs. We designed a procedure to produce ASO‐encapsulated vesicles through three steps (**Figure** **1**a): 1) To enhance the efficiency of transfecting ASO into cancer cells, the ASO were combined with a cationic konjac glucomannan (cKGM) to form cKGM/ASO complex (CKA);^[^ ^21^ ^]^ 2) the cell lines known for expressing mannose receptors (MRs) (Figure S1a, Supporting Information), receptors for cKGM, were transfected efficiently with CKA, and the modified konjac glucomannan mediated ASO transfection as efficient as did Lipofectamine 2000 (Figures S1 and S2, Supporting Information); and 3) the cells were treated with H_2_O_2_ after UV radiation to trigger apoptosis. The ABs from these cells were harvested for subsequent tests. We named the cKGM/ASO complex‐containing ABs “CABs”.

The ABs ranged in size from hundreds to thousands of nanometers.^[^ ^19^ ^]^ Interestingly, the CABs, the ASO‐loaded ABs, were generally smaller (significantly smaller than 1000 nm) (Figure 1b). Therefore, we separated these small nanoscale‐sized (<1000 nm) CABs (sCABs) from the mixture via a gradient centrifugation protocol described in Figure S3, Supporting Information. As cell apoptosis is a continuing process lasting for hours, we analyzed B16F10‐sCABs harvested at the indicated times and found that those harvested after 36 h of apoptosis had the smaller and uniform sizes and strongest Annexin V^+^ signals (a marker for ABs) (Figure S4, Supporting Information). Moreover, among the three sCABs, those derived from B16F10 were the smallest in size (Figure 1c) and had the highest Annexin V^+^ ratio (Figure S5, Supporting Information). Morphological analysis by transmission electron microscopy (TEM, Figure S6, Supporting Information) showed that the sCABs were typical cell‐derived vesicles with membrane structures. We quantified the ASO in sCABs (Figure 1d) and found that the encapsulation efficiencies of sCABs from B16F10 (21.6%) and ANA‐1 (27.4%) cells were much higher than those from 4T1 (9.1%) cells, which was consistent with the transfection efficiencies of these cells by CKA (Figure S7, Supporting Information). The higher transfection efficiency in B16F10 or ANA‐1 cells might have been due to the higher MR expression (Figure S1a, Supporting Information). Meanwhile, sABs from three cells packed more ASO than did their exosomes (Figure S8, Supporting Information). As measured, more than 10^9^ of sCABs were secreted from 1 × 10^7^ cells. Based on the sCABs numbers from a single cell (Figure 1e), we calculated the copy numbers of ASO in a single sCAB as follows: B16F10 had 1.42 × 10^15^ copies, ANA‐1 had 2.24 × 10^15^ copies, and 4T1 had 0.36 × 10^15^ copies (Figure 1f). These sCABs were quite stable, and their diameters and zeta potentials (ZPs) remained unchanged in pure phosphate‐buffered saline (PBS) and PBS supplemented with fetal bovine serum (FBS) (Figures S9, Supporting Information).

We analyzed the nucleotide and protein contents to obtain general information on sCABs. The results in Figure 1g,h and Figure S10, Supporting Information demonstrated that there was little DNA but abundant RNA in the sCABs. Little DNA was observed in monocytes‐secreted ABs with about 1 µm diameter, indicating the distribution of DNA fragments might be linked with the diameters of ABs.^[^ ^21^ ^]^ The gel electrophoresis examination in Figure 1i further shows that the RNA within sCABs had a wide size distribution. Protein liquid chromatography mass spectrometry (LC‐MS) analysis, shown in Tables S1–S3, Supporting Information, revealed more than 1000 kinds of proteins in each sCABs, and the major proteins were classified based on cellular components and their functions (Figure S11, Supporting Information). In Kyoto Encyclopedia of Genes and Genomes (KEGG) analysis, the pathways of ubiquitin proteasome and cytoskeletal regulation by Rho GTPase were more concentrated in three sCABs. To sum up, membrane proteins were abundant in all sABs, and might be crucial for the behavior of sABs inheriting from the donor cells.

Taken together, these data demonstrated that we were able to obtain massive numbers of sCABs loaded with a sufficient quantity of ASO through a simple “transfection‐apoptosis” procedure. These vesicles were small in size and were stable in serum, suggesting that they could be exploited as carriers for ASO.

### 2.2 B16F10‐sCABs Delivered ASO into Brain Microglial Cells

**Figure 2:**
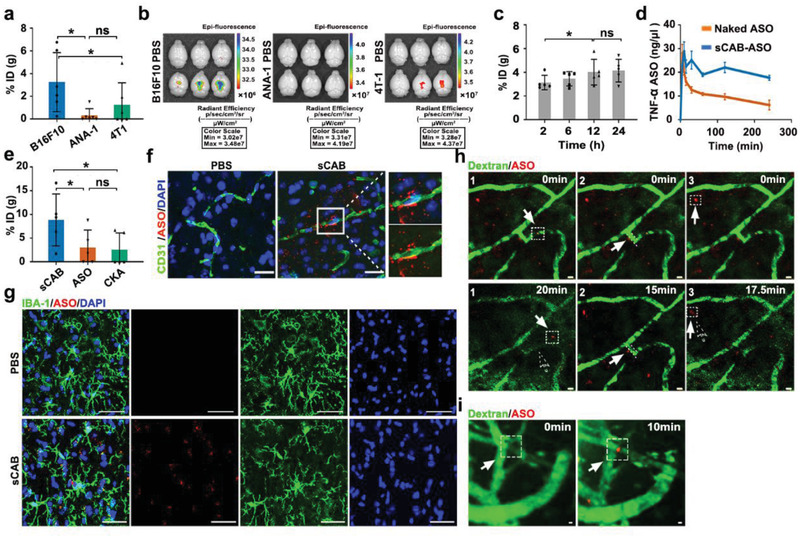
Delivering ASO into the brain by sCABs. a) Brain delivery efficiencies of the 3 types of sCABs (ID: injection dose) 24 h after the injection of 1 × 10^8^ sCABs containing 0.13 µg ASO. The %ID was calculated as the amount of ASO in the whole brain tissues to the total injection dose into mice. b) Examinations of brains harvested from mice that received 1 × 10^8^ sCABs loaded with 0.13 µg Cy5‐ASO by the IVIS Lumina XR small animal imaging system. The brains were isolated 24 h after the injection. The excitation and emission wavelengths were 640 and 670 nm, respectively. c) Time‐series analysis of ASO in the brains of mice that received 2.7 × 10^9^ sCABs with 3.5 µg ASO. d) Hemodynamic analysis of naked and sCABs‐delivered ASO at dose of 3.5 µg. e) Brain delivery efficiency of the ASO of sCABs compared to that of CKA and naked ASO. Mice were injected at the same dose of 3.5 µg ASO into tail vein and brain tissues were excised 24 h later. f) Fluorescence microscopy images of a brain section showing sCABs‐delivered Cy5‐ASO around the brain blood vessels 15 min after injection. The right panels show the magnifications of the areas selected by white square. Scale bar: 25 µm. g) Representative images from fluorescence microscopy showing Cy5‐ASO delivered by sCABs in microglial cells 20 min after injection. Scale bar: 25 µm. h). Intravital real‐time scanning showing the Cy5‐ASO delivered by sCABs in the mouse brain around the microvessels 10 min after injection. The representative tracks of sCABs during their penetration through BBB were respectively labelled and numbered as h‐1, h‐2, h‐3 in this figure, which were consistent with those in the Supporting Information Video 2. The solid arrows and the dotted boxes indicate the positions of ASO (loaded in sCABs) at the indicated times, and the dotted arrow indicates the positions at the beginning of filming (0 min). Scale bar: 5 µm. i) Representative fluorescence microscopy images showing the accumulation of Cy5‐ASO delivered by sCBAs on the abluminal side of the vessels 30 min after the injection. The arrows and dotted boxes highlight the sites where sCABs gathered over time. Scale bar: 5 µm. Images are representative of three independent experiments. Representative results are presented as the means ± SD; *n* ≥ 5 per group. *p*‐values are calculated using one‐way ANOVA followed by Bonferroni's multiple comparisons post hoc test (Figure 2a,c,e), ^*^ *p* < 0.05.

As three sABs showed little effects on b.End3 cells after 12 h incubation (Figure S12, Supporting Information), we next examined whether sCABs could deliver ASO into the brain. Three sCABs collected at 36 h after apoptosis were administered to mice via their tail vein with 1 × 10^8^ sCABs containing 0.13 µg ASO, and brain tissues were harvested 24 h after administration. **Figure** **2**a demonstrates that B16F10‐sCABs had a higher delivery efficiency (3.25%ID per g brain, ID: injection dose) than ANA‐1‐sCABs (0.32%ID per g brain) or 4T1‐sCABs (1.24%ID per g brain). These results were confirmed by an in vivo imager with an IVIS Lumina XR system (Figure 2b) and were consistent with melanoma cells having a high brain metastasis tendency.^[^ ^4^ ^]^ Based on these results, we chose sCABs from B16F10 cells as carriers in subsequent studies.

We determined the maximum tolerated dose of sCABs as 2.7 × 10^9^ particles per injection before further evaluation (Figure S13, Supporting Information). With an injection of ASO (3.5 µg) in 2.7 × 10^9^ sCABs, the ASO accumulation in the brain increased over time and peaked at 12 h post administration (Figure 2c). The results in Figure S14, Supporting Information further confirmed the time‐dependent manner of ASO accumulation in the brain, which was potentially due to the longer circulation time of sCABs (Figure 2d). Moreover, sCABs were remarkably more efficient than naked ASO and CKA (Figure 2e).

To precisely locate sCABs in the brain, cerebral microvascular endothelial cells (BMECs) along with the other three resident cells (neurons, microglial cells, and astrocytes) were labeled with Cy5‐ASO. In the parenchyma, we found that sCABs successfully passed through the microvessels (Figure 2f) 15 min after the injection and was mainly taken up by microglial cells (Figure 2g; Figure S15, Supporting Information), but not by neurons and astrocytes 20 min after the injection (Figure S16, Supporting Information).

**Figure 3:**
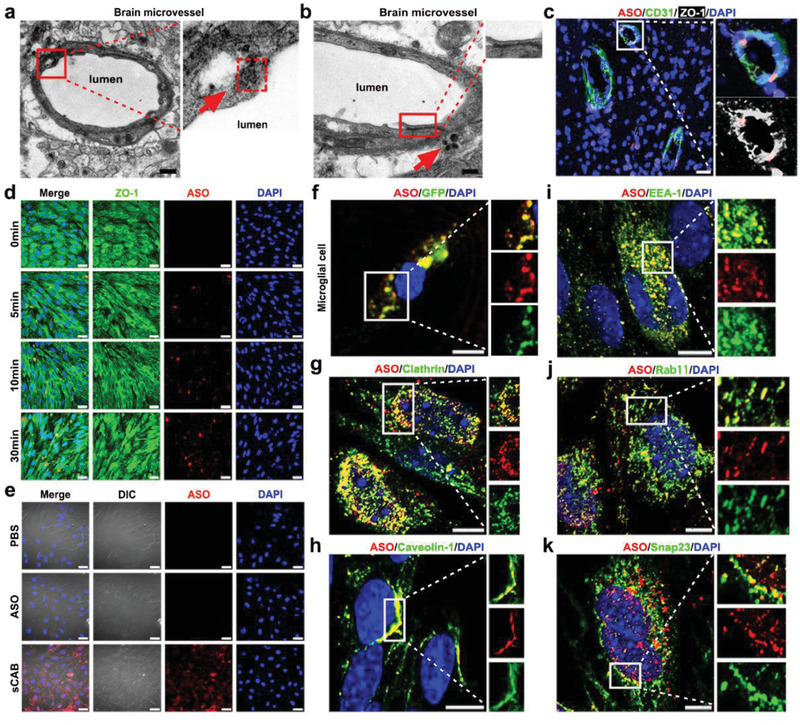
sCABs are transcytosed by BMECs to cross the BBB. a) TEM images showing a phagosome containing a sCAB in the BMEC of a mouse 10 min after an i.v. sCABs injection. The right panel shows a magnified view of the phagophore selected by the red square and arrow. Scale bar: 500 nm. b) TEM images showing the structure of TJs after sCABs passed through microvessels 15 min after an i.v. sCABs injection. The TJs in the red squares are shown at high magnifications on the right. The red arrow depicts sCABs that passed through BMECs. Scale bar: 500 nm. c) Representative fluorescence images showing the intact structure of TJs after sCABs treatment containing Cy5‐ASO. The right panels show magnified views of the area selected by the white square. Scale bar: 25 µm. d) Representative fluorescence microscopy images showing sCABs containing Cy5‐ASO phagocytized by b. End3 cells in the BBB model. Scale bar: 25 µm. e) Representative fluorescence images showing Cy5‐ASO delivered by sCABs but not the naked one was phagocytized by microglial cells in the BBB model 24 h after the incubation. The morphology of microglial cells as imaged with differentia linterference contrast (DIC). Scale bar: 25 µm. f) Fluorescence images showing GFP‐labeled sCABs containing Cy5‐ASO phagocytized by microglia 24 h after the incubation. The right panels show magnifications of the area selected by the white square. Scale bar: 10 µm. g–k) Representative fluorescence images showing the colocalization of sCABs containing Cy5‐ASO with special protein markers ((g) labeled with clathrin, (h) with caveolin‐1, (i) with EEA‐1, (h) with Rab11 involved in endocytosis, and (k) with Snap23). The colocalization of clathrin and caveolin‐1 was examined at 5 min after sCABs incubation, EEA‐1 and Rab11 at 10 min, and Snap23 at 20 min. The right panels show magnifications of the areas selected by the white square. Scale bar: 25 µm. Images are representative of three independent experiments.

To directly observe the process of sCABs passing though the BBB, we used a two‐photon confocal microscopy system (LSM 980 NLO with Airyscan2) for intravital real‐time observation.^[^ ^22^, ^23^, ^24^ ^]^ The skull was thinned to enable a good view window, and the vessels were visible with the intravenous injection of dextran (Video S1, Supporting Information).^[^ ^24^, ^25^, ^26^ ^]^ After the injection of 2.7 × 10^9^ sCABs containing 3.5 µg Cy5‐ASO, the scanning was conducted every 220 ms for 20 min (Figure 2h; Video S2, Supporting Information) 10 min later. First, we observed a process in which the free‐floating sCABs in microvessels adhered to the inner surface of the vessel before they presented in the parenchyma outside the blood vessel (Figure 2h‐1). To reach the parenchyma, sCABs must pass through more barriers, including the basement membrane, pericytes, and astrocyte feet, in addition to tight junction (TJ)‐characterized BMECs. The slow movement of sCABs away from the abluminal side of the vessels in Figure 2h‐2 might be related to this process. Third, a sCAB signal in the parenchyma (Figure 2h‐3) moved ≈5 µm within 20 min before microglial cells were taken up. Additionally, the process of sCABs accumulation on the abluminal side of the vessels was monitored (Figure 2i; Video S3, Supporting Information).

Together, these data demonstrated that sCABs were capable of efficiently delivering ASO across the BBB and into microglial cells.

### 2.3 sCABs Transcytosed by Endothelial Cells of the BBB

A carrier can penetrate the BBB via two possible mechanisms: 1) By breaking TJs and bypassing endothelial cells,^[^ ^27^, ^28^ ^]^ or 2) by being phagocytosed and then secreted by endothelial cells without interacting with TJs.^[^ ^12^, ^22^, ^29^ ^]^ To determine which mechanism was utilized by sCABs, we first examined the BMECs in brain sections 10 min after sCABs administration (3.5 µg ASO in 2.7 × 10^9^ particles) with TEM and discovered that they resided in the phagosomes of BMECs (**Figure** **3**a) and exhibited a membrane structure similar to that observed in vitro (Figure S17, Supporting Information). Meanwhile, no obvious structural change in TJs was found (Figure 3b,c). These results suggested that sCABs were phagocytosed by BMECs instead of breaking the TJs.

Phagocytosed by BMECs, drugs must undergo exocytosis to reach the parenchyma, which is termed a “transcytosis” process. We then constructed an in vitro BBB model (Figure S18, Supporting Information) in which the primary astrocytes extracted from mice were characterized with glial fibrillary acidic protein (GFAP) (Figure S19a, Supporting Information).^[^ ^30^, ^31^, ^32^ ^]^ During a two‐week coculture with astrocytes, the TJs by the mouse BMECs were evidenced by the transepithelial/transendothelial electrical resistance (TEER) value, an indicator for TJs (Figure S19b, Supporting Information) and marked with zonula occluden‐1 (ZO‐1), which contributed to electrical resistance (Figure S19c, Supporting Information).^[^ ^33^ ^]^ Primary microglial cells were verified to express ionized calcium bindingadaptor molecule‐1 (IBA‐1) (Figure S19d, Supporting Information) and were 89% positive for CD11b according to the gating strategy shown in Figure S19e, Supporting Information. Two weeks later, we replaced astrocytes with microglial cells in the bottom chamber to establish the in vitro BBB model.

The incubation of sCABs (65 ng ASO delivered by 5 × 10^7^ particles) with the b. End3 cells did not damage the TJs (Figure 3d; Figure S20, Supporting Information), while sCABs were found on the bottom sides of microglial cells 24 h later (Figure 3e), suggesting that they were released from endothelial cells after endocytosis. We further evaluated whether the ASO was released in the sCABs as a whole during the process. We labeled the sCABs membrane with GFP by using GFP‐transgenic B16F10 cells, as shown in Figure S21, Supporting Information. The colocalization of GFP and ASO in microglial cells illustrated that sCABs completed transcytosis though b. End3 cells as unchanged vesicles (Figure 3f).

According to the reported mechanisms, three major steps make up one transcytosis process: 1) Endocytosis on the apical membrane of BMECs, 2) intracellular trafficking, and 3) release into the extracellular environment at the basolateral membrane.^[^ ^34^ ^]^ We monitored the whole process and first detected the colocalization of Cy5‐ASO with caveolin‐1 and clathrin 5 min after the incubation, indicating that caveolin‐ and clathrin‐dependent endocytosis was involved in initiating transcytosis (Figure 3g,h). Then, the ASO was colocalized with early endosome antigen‐1 (EEA‐1), a marker of early endosomes, and Ras‐related protein (Rab11), an indicator for recycling endosome (Figure 3i,j) 10 min after the incubation, demonstrating that sCABs were transferred into early and recycling endosomes later, avoiding lysosome degradation. Finally, we found that soluble NSF attachment protein 23 (Snap23), a factor involved in the interaction of endosomes with the basolateral membrane, colocalized with the Cy5‐ASO (Figure 3k) 20 min after the incubation, marking the release of sCABs from endothelial cells.

Together, the above data and analysis demonstrated that sCABs were first transferred into early endosomes and then into Rab11^+^ recycling endosomes after they were taken up by endothelial cells and eventually released from the basolateral membrane of the cells.

### 2.4 CD44v6 Mediated the Transcytosis of sCABs Through BMECs

**Figure 4:**
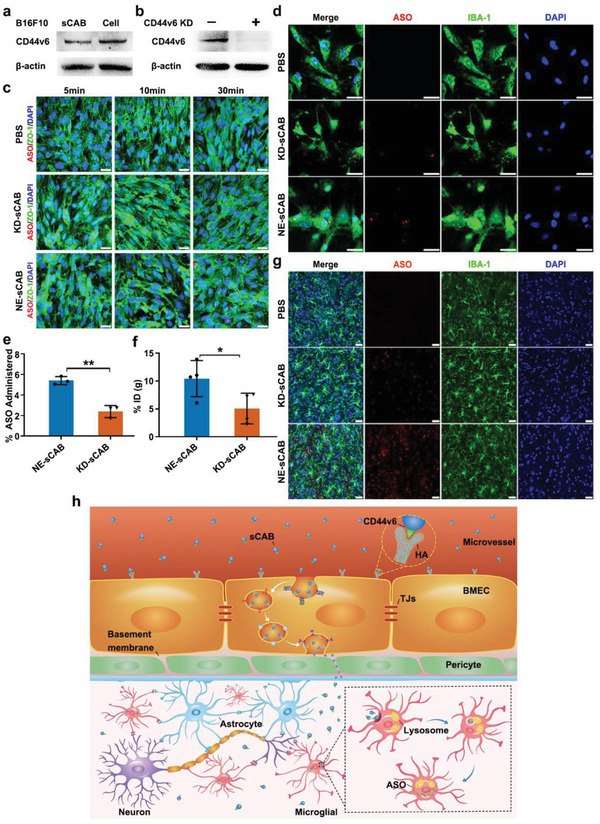
CD44v6 mediated the transcytosis of sCABs through BMECs. a) Western blot showing the expression of CD44v6 in B16F10 cells and in sCABs. b) Western blot showing reduced expression of CD44v6 in sCABs from CD44v6 knockdown B16F10 cells. CD44v6 KD: CD44v6 Knockdown. c) Representative fluorescence images showing Cy5‐ASO delivered by different sCABs taken up by b. End3 cells in the BBB model. KD‐sCAB: sCABs from CD44v6 knockdown B16F10 cells; NE‐sCAB: sCABs from normal B16F10 cells. Scale bar: 25 µm. d) Representative images showing Cy5‐ASO in microglial cells in the BBB model 24 h after the incubation with sCABs. Scale bar: 25 µm. e) The ASO delivery efficiencies of NE‐ and KD‐sCABs to microglial cells in the BBB model. f) The efficiencies of NE‐ and KD‐sCABs for delivering 3.5 µg ASO by 2.7 × 10^9^ particles into brain microglial cells 24 h after the injection. g) Representative images showing Cy5‐ASO delivered by NE‐ and KD‐sCABs into the brain 30 min after the injection. Scale bar: 25 µm. h) Schematic diagram of sCABs transcytosed by BMECs. Images are representative of three independent experiments. Representative results are presented as the means ± SD; *n* ≥ 3 per group. *p*‐values are calculated using Student's *t*‐test (Figure 4e,f), ^*^ *p* < 0.05, ^**^ *p* < 0.01.

As reported, members of the CD44 protein family, particularly its variant isoforms, play critical roles in tumor cells metastasis and immune cell infiltration into the brain.^[^ ^35^, ^36^ ^]^ CD44v6, a splice variant of the CD44 protein, is highly expressed on cells with strong brain metastasis potential.^[^ ^7^, ^35^, ^37^ ^]^ We studied whether CD44v6 was also the key mediator for the transBBB delivery of sCABs. Consistent with the delivery efficiency trends, CD44v6 was more abundantly expressed in B16F10 cells than in ANA‐1 or 4T1 cells, and more importantly, it was also expressed at higher levels in the B16F10‐sCABs than in the ANA‐1‐ or 4T1‐sCABs (**Figure** **4**a; Figure S22, Supporting Information). To prove that CD44v6 was a critical cell adhesion molecule (CAM) for sCABs, we knocked CD44v6 down in B16F10 cells by ≈50% (Figure S23, Supporting Information) and in sCABs (Figure 4b). The morphology, Annexin V^+^ ratio and ASO packing ability of the sCABs with decreased CD44v6 expression were not different from those of the sCABs expressing normal levels of CD44v6 (Figure S24, Supporting Information). Then, we analyzed the delivery efficiency of CD44v6‐knockdown sCABs in vitro and in vivo. In the BBB model, ASO at dose of 65 ng delivered by 5 × 10^7^ sCABs was incubated with b.End3 cells. Fewer CD44v6‐knockdown sCABs were phagocytized by b.End3 cells (Figure 4c), and their efficiency for delivering ASO to microglial cells was also reduced (Figure 4d,e). Consistently, the CD44v6‐knockdown sCABs (3.5 µg ASO delivered by 2.7 × 10^9^ particles) displayed a decreased efficiency for delivering ASO to the brain (Figure 4f,g; Figure S25, Supporting Information).

In summary, these data suggested that the recognition and binding of CD44v6 in sCABs to BMECs play a critical role in transcytosis. This process is summarized in Figure 4h.

### 2.5 Anti‐TNF‐*α* ASO delivered by sCABs Reduced Brain Inflammation and PD Symptoms in Mice

Hemagglutinin (HA) is upregulated by inflammatory stimulators together with the primary adhesion of CD44/HA.^[^ ^38^ ^]^ We found that lipopolysaccharide (LPS)‐treated microglial cells took up more sCABs than nontreated cells (Figure S26, Supporting Information). This effect also occurred in vivo, as the accumulation of ASO in brains with intrabrain LPS administration peaked much quicker (4 h postadministration) than that in normal mice (12 h postadministration) (Figure S27, Supporting Information). Meanwhile, the kinetics and distribution of Cy5‐ASO delivered by sCABs in PD models were different from those in normal mice. PD models had an increased accumulation of the Cy5‐ASO (with or without the carriers) in the brain, spleen, and kidney but a decreased amount in the liver and lung. The reason might be the influence of LPS injected into the brain, which probably impacted the metabolism of the physiological system. These phenomena suggested that sABs are an ideal carrier for combating inflammation in the brain, which was verified first in vitro. In our experiments, sCABs treatment efficiently downregulated TNF‐*α* expression and reduced morphological abnormities in microglial cells stimulated by LPS (Figure S28, Supporting Information).

**Figure 5:**
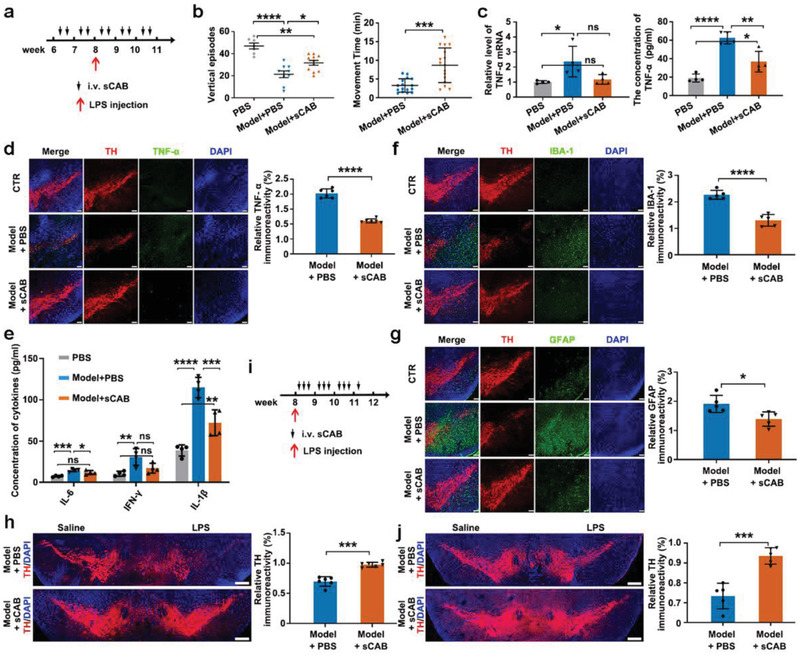
Effects of sCABs treatment on PD model mice. a) The schedule protocol for sCABs (3.5 µg ASO delivered by 2.7 × 10^9^ particles per injection) preventative treatment in PD mice. b) Testing the vertical episodes and movement times of the mice. c) TNF‐*α* mRNA and protein levels in mice receiving the treatments. d) Representative fluorescence images showing the expression of TNF‐*α* in the brains of mice that received different treatments and statistical analysis of the fluorescence intensity. Scale bar: 100 µm. e) Quantification of the three proinflammatory cytokines in the mouse brains after the treatments. f,g) Representative fluorescence images showing the morphological changes in microglial cells (f) and astrocytes (g) and statistical analysis of the fluorescence intensity. Scale bar: 100 µm. h) Representative fluorescence microscopy images of TH^+^ dopaminergic neurons in the SNc area and statistical analysis of the fluorescence intensity. Scale bar: 250 µm. i) The treatment schedule post PD establishment and sCABs were injected at dose of 2.7 × 10^9^ particles for delivering 3.5 µg ASO for 10 times. j) Representative fluorescence images showing the TH^+^ dopaminergic neurons in the SNc and statistical analysis of the fluorescence intensity. Scale bar: 250 µm. Images are representative of three independent experiments. Representative results are presented as the means ± SD; *n* ≥ 4 per group. *p*‐values are calculated using one‐way ANOVA followed by Bonferroni's multiple comparisons post hoc test (vertical episodes in Figure 5b,c,e) and using Student's *t*‐test (movement times in Figure 5b,d,f,g,h,j), ^*^ *p* < 0.05, ^**^ *p* < 0.01, ^***^ *p* < 0.001, ^****^ *p* < 0.0001.

Encouraged by the in vitro anti‐inflammatory effects, we further tested whether sCABs were efficacious in the treatment of PD in a mouse model induced by intra‐substantia nigra pars compacta (SNc) LPS injection.^[^ ^39^, ^40^ ^]^ The prevention test was conducted as shown in **Figure** **5**a. Briefly, mice were given sCABs (3.5 µg ASO delivered by 2.7 × 10^9^ sCABs per injection) 4 times *iv*. before the initiation of PD, followed by another 6 treatments. According to the results shown in Figure 5b and Figure S29, Supporting Information, the mice treated with sCABs had improved vertical movement episodes and total movement times and ameliorated climbing time, which suggested relief of PD development.^[^ ^41^ ^]^ Further, compared with those in untreated mouse brains, the TNF‐*α* mRNA levels and protein levels in the brains of sCABs‐treated mice were decreased by 50% and 41%, respectively (Figure 5c). Consistently, the fluorescence intensity (FI) of TNF‐*α* in the SNc areas was decreased by ≈45% (Figure 5d). The proinflammatory IFN‐*γ*, IL‐1*β*, and IL‐6 were also significantly lowered in the brain tissues after sCABs treatment (Figure 5e), and the numbers of activated microglial cells and astrocytes were both decreased by ≈39% (Figure 5f,g; Figure S30, Supporting Information). Importantly, as the most important marker of PD development, the survival of dopaminergic (DA) neurons (labeled as tyrosine hydroxylase‐positive (TH^+^)) was improved by ≈40% with sCABs treatment (Figure 5h; Figure S31, Supporting Information). In general, after sCABs treatment, the level of TNF‐*α* secreted by microglial cells was lowered, as well as the other pro‐inflammatory factors. The numbers of activated microglial cells and astrocytes were both decreased accordingly. With meliorative inflammation in brains, the viability of neurons improved followed with the ameliorative PD symptom.

Next, we examined the effects of sCABs on PD treatment post disease initiation. Mice were treated with sCABs (3.5 µg ASO delivered by 2.7 × 10^9^ sCABs per injection) every 2 days 10 times after PD induction, as shown in Figure 5i. Then, their symptoms were analyzed with the same methods as those used in the PD prevention tests. In the behavioral evaluation, improved vertical movement episodes, total movement times, and ameliorated climbing time were observed (Figure S32, Supporting Information). The levels of TNF‐*α* and other inflammatory cytokines were lowered after treatment (Figures S33 and S34, Supporting Information). The improvement in the survival of DA neurons was ≈28% (Figure 5j, Figure S35, Supporting Information), and the numbers of activated microglial cells and astrocytes were decreased by 36% and 41%, respectively (Figures S36 and S37, Supporting Information). To assess the safety of the treatment, different organs from mice treated with B16F10 sCABs for 10 times went for a histopathological examination by hematoxylin‐eosin (H*&*E) staining 6 months later and no significant abnormities were found (Figure S38, Supporting Information).

Together, these experiments suggested that sCABs efficiently suppressed TNF‐*α* in the PD mouse brain and significantly ameliorated the symptoms of PD, suggesting that sABs have the potential to be a brain‐targeting delivery carrier for the treatment of central nervous system diseases such as PD.

## 3 Discussion

Crossing the BBB remains one of the toughest challenges in drug delivery research. However, many cancer cells have the capacity for brain metastasis, suggesting that these cells exploit particular mechanisms to hack into the brain.^[^ ^4^, ^5^ ^]^ Although the precise mechanism underlying the process is still under investigation, some key molecules have been identified to facilitate the intrabrain invasion of tumor cells. According to recent studies, CD44v6 is essential for cancer brain metastasis and specifically binds to polysaccharides on the BBB.^[^ ^7^, ^35^, ^36^, ^37^ ^]^ Based on this finding, we designed a strategy to biologically produce vesicles in which CD44v6 was integrated into their membrane and tested whether they could mediate the intrabrain delivery of an ASO drug to suppress brain inflammation in PD mouse models. As our results showed, the sABs expressing CD44v6 had a remarkably high efficiency for penetrating the BBB, undergoing a transcytosis process in which they passed though the endothelial cells intact. This phenomenon is interesting and different from the brain metastasis of melanoma cells.

In our study, B16F10 sCABs showed the higher delivery efficiency for ASO than those of ANA‐1 and 4T1. Clinically, even though the frequency of brain metastasis of melanoma is ranked behind breast cancers and some glandular cancers, melanoma showed the highest tendency for brain metastasis in epidemiologic study during decades. The ratio of brain metastasis is as high as 50–60%. It might be an explanation for the highest delivery efficiency of B16F10 sABs. Besides, the expression of CD44v6 protein in B16F10 cells and their sABs was much higher than that in ANA‐1 and 4T1. So, in our opinion, CD44v6 is the critical factor for brain metastasis. Interestingly, the B16F10 sABs were also much more uniform than the others, which probably contributed to their higher transport efficiency.

Unlike conventional exosomes, ABs are rarely considered for drug delivery, due to their uneven size distribution (varying from hundreds to thousands of nm), complex content (especially large chromosomal DNA fragments and various kinds of cytoplasmic proteins), and potential apoptosis‐inducing activities.^[^ ^42^, ^43^ ^]^ Moreover, ABs are easily cleared by phagocytes, adding more questions on their use for drug delivery. However, according to our analysis in the present study, the vesicles produced by apoptotic cells probably do not belong to the same category. They could be roughly divided into two categories by their size: large (larger than 1 µm) and small (≈100–1000 nm). The differences in diameters may suggest that the small “ABs” might not be of the classical type, although they were also Annexin V^+^. Annexin V is a common molecule for PS testing as it is recognized and bounded by reversed PS on the membrane of ABs. PS is the typical “eat me” signal of ABs to be phagocytosed by microglial cells. In other words, the high ratio of Annexin V indicated that more ABs containing ASO would be phagocytosed by microglial cells and regulated the inflammatory response in brains. According to their characteristics, they could be a specific type of shedding vesicle generated during the apoptosis process and play a role different from that of classic ABs, mainly as residues of dead cells.

Our data further demonstrated that these small bodies possess some properties that are not consistent with our knowledge about ABs. First, they contained no chromosomal DNA fragments, which is the most typical characteristic of classic ABs. Second, they are stable in serum and demonstrated a long circulating time when injected in vivo, meaning that they were not readily recognized and engulfed by phagocytes. These two properties indicate that these small bodies do not function as classic ABs to preserve the cell components from apoptotic cells but rather as special messengers, like exosomes, carrying a large amount of RNA and circulating in the blood. Considering that apoptosis is also a typical cellular response to internal or environmental stimuli, secreting vesicles with specific information to inform other cells in the body is reasonable and necessary for maintaining the stability of a physiological system. On the other hand, these special properties prepare sABs as potential EV‐based drug carriers with a small size, long circulation times, and stealth capacity.

The sABs have several important advantages that make them a better candidate for drug delivery. It is possible to overcome the main challenges in the development of drug delivery systems based on exosomes or other EVs. First, the cell apoptosis process is much more controllable than the biogenesis of exosomes, thanks to our more extensive understandings into cell apoptosis than that of exosomes or other EV biogenesis procedures. Better understanding leads to a more controllable manufacturing, which is important for scaling up the delivery system for pharmaceutical applications. With mature apoptosis‐inducing technology, the quantity of sABs can be readily expanded in a pharmaceutical industrial setting, and batch‐to‐batch differences can also be minimized via standard operating procedures. Second, the efficiency of drug loading into sABs is much higher than that into exosomes, as cell apoptosis is a process that completely divides the cell into pieces, meaning that all cytoplastic contents are mandatorily enveloped into vesicles generated from dying cells. This concept was also proven by our results of the entrapment efficiency of ASO into small bodies being quite high and comparable to that of artificial liposomes. Additionally, the loading of drugs into sABs is simpler than that into exosomes, as a series of mechanisms—including how cells are packaged into exosomes and which factors determine which molecules for packaging are poorly understood, due to the lack of basic knowledge about exosome biogenesis.^[^ ^44^ ^]^ Finally, the sABs are vesicles shed from the cell membrane, and the molecules in the cell membrane are preserved on the surface of the vesicles, which is another advantage for incorporating targeting ligands.^[^ ^16^, ^17^ ^]^

Our findings support a recent study that appreciates the cell membrane of ABs as new drug delivery system.^[^ ^17^ ^]^ The researchers elegantly utilized ABs for their natural targeting for macrophages and anti‐inflammatory activity, both based on the membrane proteins on the ABs. Notably, the chemotaxis of ABs inheriting from the active T cells could guide the drugs to the inflammation sites. In both their and our strategies, the utilization of natural characters of the ABs without other artificial biochemical conjugation could avoid unwanted changes to the structures or functions of ABs. Meanwhile, the intracellular stuff was removed from the ABs, increasing the space for loading drugs.

In our study, CD44v6 expressed by melanoma cells was abundantly present on the surface of sABs and functioned as the key mediator of cross‐BBB delivery. Taken together, these results indicate that exploiting sABs as new EV carriers for in vivo drug delivery is feasible and has significantly higher clinical relevance than other EVs.

Another intriguing finding is that apoptotic cells seem to specifically package RNAs into sABs. The single strand ASO used in our study might be differentiated as RNA molecules by apoptotic cells when packaging materials into these vesicles. This phenomenon suggests that the efficiency of loading and delivering siRNAs or microRNAs would be even more efficient.

However, to advance the technology into clinical practices, especially for the treatment of brain disease, safety concerns should be more carefully assessed. Although no notable adverse effects were detected in our study, the possibility of vesicles triggering unwanted cellular responses should be studied in future work. To investigate this, special sABs should be considered as a new type of vesicle, and more studies should be carried out to explore their natural functions.

## 4 Experimental Section

##### Reagents and Synthesis of Materials

The ASO ISIS‐25302 (base sequence: 5′‐AACCCATCGGCTGGCACCAC‐3′) against mouse TNF‐*α* was synthesized by Saibai Sheng Company (China) with phosphorothioate modification for all nucleotides. FAM‐ and Cy5‐labeled ASO were applied for cellular localization and tissue distribution.

KGM was obtained from Megazyme (Wicklow, Ireland), and cKGM was prepared as previously reported.^[^ ^22^ ^]^ Briefly, ethylenediamine was introduced to the hydroxyl groups of KGM after *N,N′‐*carbonyldiimidazole (CDI) activation. The cationic degrees were determined with different ratios between the hydroxyl groups of KGM and CDI, and the mole ratio was selected as 1:5 in this study. Lipofectamine 2000 (Cat. No. 11668‐019) was provided by Invitrogen, Thermo Fisher Scientific. LPS (L2630) was purchased from Sigma (USA). The other chemical agents and enzymes were obtained from Sangon Biotech (China).

##### Cells Culture

B16F10 cells, b. End3 cells, 293T cells, primary microglial cells, and astrocytes were cultured in DMEM, while ANA‐1 and 4T1 cells were cultured in complete RPMI medium. The cell lines (ATCC, USA) were supplemented with FBS (10%) and penicillin/streptomycin and grown at 37 °C in 5% CO_2_ incubators (Thermo Fisher Scientific, USA). The culture media and FBS were obtained from Gibco (Thermo Scientific, USA).

##### Mice

Male C57BL/6J mice (weighing ≈18–20 g) were purchased from the Experimental Animal Center of Nanjing Medical University (Nanjing, China). The mice were housed in specific pathogen‐free isolators under controlled conditions regarding food, water, light, and humidity. All animal protocols were approved by the Animal Care and Use Committee of Nanjing University and conformed to the Guidelines for the Care and Use of Laboratory Animals published by the National Institutes of Health. The Number of Animal Use Permit was SYXK(Su)2019‐0056.

##### Collection and Characterization of sCABs

CKA produced by mixing TNF‐*α* ASO (0.33 mg mL^−1^) and cKGM (5 mg mL^−1^) in sterile phosphate buffer (pH 7.0) at a mole ratio of 5:1 for 30 min in sterile phosphate buffer (pH 7.0). The complexes containing 165 µg ASO were then incubated with 1 × 10^7^ cells for 6 h. Then, the old medium was replaced with serum‐free DMEM/1640 supplemented with H_2_O_2_ (400 nм) after 30 min of UV at 150 mJ cm^−2^ radiation to induce the apoptosis.

The medium was collected from CKA treated cells 36 h after apoptosis induction and sCABs were separated by gradient centrifugation as described in Figure S3, Supporting Information. Briefly, the dead cells and debris were discarded after centrifugation at 300 g × 10 min, following the separation of bigger ABs at 3000 g × 20 min. The supernatant was further centrifugated at 12 000 g × 30 min to collect sCABs in the sediments that were washed with PBS (pH 7.0) for three times. Then, the sCABs resuspended in phosphate buffer (pH 7.0) were counted and analyzed for diameter by a nanoparticle tracking analysis (NTA, NanoSight NS300, Malvern, UK). The ZP was monitored with a Nano‐Z instrument (Malvern, UK). The Annexin V^+^ ratios for sCABs were analyzed by flow cytometry (Attune NxT, Invitrogen, USA) with a FITC‐Annexin V apoptosis detection kit (bs‐0450R, Bioss, China).

##### TEM Analysis of sCABs

The sCABs pellets were resuspended in paraformaldehyde (PFA, 2%). TEM (Tecnai G2 Spirit Bio TWIN, Thermo Fisher Scientific, USA) was applied to observe the morphology of sCABs stained with a uranyl‐oxalate solution (pH 7.0) and with methyl cellulose‐UA at 120 kV by EM.^[^ ^45^ ^]^

##### Transfection Efficiency Analysis of cKGM/ASO Complex

The naked ASO or CKA containing 5 µg ASO were incubated with 2 × 10^6^ B16F10 cells for 6 h. Meanwhile, the transfection of the Lipofectamine 2000/ASO complex was performed as a control according to the manufacturer's protocol. Then, the medium was removed and cells were rinsed with PBS for three times. To quantify the transfection efficiency, transfected cells were collected by an RNA isolation protocol and analyzed via quantitative real‐time polymerase chain reaction (RT‐qPCR) to determine quantity of the ASO. The transfection efficiency was calculated as the ratio of intracellular ASO to the amount of ASO added into the well.

##### Isolation of Exosomes

For the isolation of exosomes, three cell lines at 1 × 10^7^ were cultured in 150 cm dishes and CKA containing 165 µg ASO were added into the dishes and incubated for 6 h. After incubation, cells were washed with PBS for three times. Fresh medium was added into the cells. The cells were cultured for another 72 h for exosomes release. The medium was then collected and separated for exosomes by gradient centrifugation.^[^ ^46^ ^]^ ASO in exosomes were isolated via the TRIzol assay and then analyzed with RT‐qPCR as mentioned above.

##### Cell Viability Assay

Cytotoxicity of sABs was examined with the cell counting kit‐8 (CCK‐8, Dojindo, Tokyo, Japan). The b.End3 cells were seeded in a 96‐well plate at a number of 1 × 10^4^ cells per well and cultured overnight. Then the cells were incubated in 100 *μ*L serum‐free medium containing 1 × 10^6^ sABs from B16F10, ANA‐1, and 4T1 cells. After 12 h treatment, the medium was removed and the cell viability were analyzed with CCK‐8 assay according to the manufacture's instruction.

##### Analysis of Nucleic Acids and Proteins in sCABs

The nucleic acids and proteins in sCABs were obtained with TRIzol according to the manufacturer's instructions. After the enzymatic hydrolysis reaction with DNaseI (CW2090s, CWBIO, China) and RNaseA (EN0531, Thermo Scientific, USA) separately, the concentrations of RNA and DNA were analyzed based on the absorbance at OD_260_ by the NanoDrop One instrument (Thermo Fisher Scientific, USA). The samples of RNA and DNA were then separated by urea‐denatured polyacrylamide gel electrophoresis (5%) and a 1% agarose gel. The gels were imaged with a UV transilluminator (Gel Doc 2000, Hercules, USA) after ethidium bromide (0.1%) staining.

The proteins were washed with guanidine hydrochloride (0.3 м) at least 2 times and resuspended in sodium dodecyl sulfate (SDS) (200 *μ*L, 1%). The samples were then subjected to LC‐MS analysis on a Shimadzu UFLC 20ADXR HPLC system connected in line with an AB Sciex 5600 Triple TOF mass spectrometer (AB SCIEX, USA). The proteins were analyzed by searching the mouse taxon of the UniProtKB/SwissProt database (release 2011_11). Gene ontology (GO) (http://geneontology.org/) was used to classify the proteins based on cellular components and molecular functions. The proteins were clustered according to pathway terms identified from KEGG database.

##### Absolute Quantitative Real‐Time qPCR Analysis of ASO

ASO in tissues, sCABs or exosomes were isolated with TRIzol according to the manufacturer's instructions. After reverse transcription, RT‐qPCR was carried out on a 7500 Sequence Detection System (Applied Biosystems) with LightCycler FastStart DNA Master SYBR Green I (Roche Diagnostics, IN) according to the protocol provided by the manufacturer. The RT primers for reverse transcription and primer set for RT‐qPCR analysis are listed in Table S1, Supporting Information.

The standard curve for ASO was generated with commercial standards, as was the average recovery rate of ASO extracted from tissues, sCABs or exosomes with TRIzol. The copy number of ASO was calculated with the formula: *y = kx+b*, where *y* represents the CT value, *x* value represents the log value (copy number), *k* is related to the amplification efficiency, and the *b* value is a constant.

Then, the concentration of extracted ASO was calculated based on the copy number, and the amount of ASO in the tissues, sCABs, or exosomes was calculated by recovery rate.

The concentration of ASO (ng *μ*L^−1^) = copy number × (molecular weight) × 6.02 × 10^−14^.

##### Tissue Distribution Assay of ASO Delivered by sCABs

The sCABs containing ASO or fluorescence labeled ASO were injected via tail vein. Mice were anesthetized at indicated time points after injection and perfused with cold PBS. The brain tissues from sCABs (containing Cy5‐ASO) treated mice were excised for fluorescence imaging by an in vivo imager (IVIS Lumina XR system I, PerkinElmer, USA). The excitation and emission spectra were 640 and 670 nm, separately. Furthermore, the brain tissues were used for ASO isolation and the quantification of ASO levels by RT‐qPCR test. The %ID was calculated as the amount of ASO in the whole brain tissues to the total injection dose into mice. To determine the blood ASO level, serum was separated from blood collected from mice treated with sCABs‐delivered FAM‐ASO at indicated time points. Then, the FI of the serum was measured with a multimode reader (Varioskan LUX, Thermo Scientific, USA) and the serum ASO level was calculated according to the standard curve made by FAM‐ASO standards.

##### Tissue Preparation for Immunofluorescence/Immunohistopathological Staining and Extensive Transmission Electron Microscopy Imaging

For immunofluorescence staining, mice with different treatments were anesthetized and perfused with cold PBS and 4% PFA. The brain tissues were removed as soon as possible and fixed with 4% PFA overnight at 4 °C. Then, the brains were soaked in a constant gradient dehydration solution with sucrose and embedded with opti‐mum cutting temperature compound (OCT) for 30 min at 4 °C. The brains were sliced (25 µm in sickness) with a freezing microtome (CM1950, Leica, Austria).

The brain sections were dipped in 0.3% Triton for membrane perforation and then blocked with 5% bovine serun albumin (BSA) for 1 h at room temperature (RT). The sections were incubated with the following primary antibodies overnight at 4 °C: An antibody against IBA‐1 (CN.019‐10741, Wako, Japan), an antibody against TH (ab76442, Abcam, UK), an antibody against GFAP (ab53554, Abcam, UK), an antibody against CD31 (ab24590, Abcam, UK), and an antibody against ZO‐1 (PB9234, Boster, China). The corresponding fluorescent secondary antibodies (Life Technology, USA) were incubated with the sections after three washes. After staining with 4,6‐diamidino‐2‐phenylindole (DAPI) (C1002, Beyotime, China), the sections were imaged with a two‐photon laser confocal microscope (TCS SP8‐MaiTai M, Leica, Austria).

Immunohistochemistry (IHC) staining was carried out according to the standard protocol. The sections were incubated with a primary anti‐TH antibody over night as previously described and then an horseradish peroxidase (HRP)‐conjugated secondary antibody (ab6753, Abcam, UK) for 1 h at RT. The chromogenic system was detected with a Diaminobezidin (DAB) Color Development Kit (AR1000, Boster, China) and stopped with 3% H_2_O_2_. The images were captured with an Eclipse Ni‐E upright microscope (Nikon, Japan).

For extensive TEM imaging, mice were anesthetized after the injection of 2.7 × 10^9^ sCABs containing 3.5 µg ASO. Then, anesthetized mice were irrigated with cold PBS containing heparin sodium for 30 min and then with cold 2.5% glutaraldehyde for fixation. The brains soaked in glutaraldehyde were cut into 1 mm^3^ sections as soon as possible and fixed for at least 24 h at 4 °C. The brain sections were dehydrated and then sliced 50 nm in sickness.^[^ ^47^ ^]^ After stained with 7% uranyl acetate diluted in methanol for 12 min and lead citrate for 8 min consecutively. A Philips C12 transmission electron microscope was used at 80 kV for sample analysis.

##### Intravital Real‐Time Observation of sCABs Crossing the BBB

To directly observe the movement of the sCABs through the BBB, two‐photon confocal microscopy (LSM 980 NLO with Airyscan2, Zeiss LSM980, Germany) was used for intravital real‐time scanning. The skulls were thinned with a dental engine to extend the viewing window while the mice were anesthetized and fixed on an adaptor (68030, RWD, China). The vessels were visible with the intravenous injection of dextran (10 KD, T18988, TargetMol, USA). The real‐time extravasation of sCABs containing Cy5‐ASO was evaluated based on the intensities and distributions of fluorescent signals. After the injection of sCABs (2.7 × 10^9^ particles containing 3.5 µg), images were captured 10 or 30 min after injection and the scan was conducted every 120 ms for 20 min and videoed with Zeiss ZEN Blue 3.1 software.

##### Preparation of the BBB Model

We designed a triculture BBB model in a 24‐well transwell (140627, Thermo Fisher Scientific, USA) with a porous membrane (membrane thickness = 8 µm; pore size = 3 µm). The constitution of BBB model is shown in Figure S18, Supporting Information.^[^ ^48^ ^]^ The primary astrocytes and microglial cells were obtained from the gray matter of neonatal mice.^[^ ^30^, ^40^ ^]^ The astrocytes were then analyzed with GFAP and the microglial cells with IBA‐1, as well as a FITC‐CD11b antibody (101205, eBioscience, USA) by flow cytometry. b. End3 cells were first seeded on the apical side of the permeable filter at 5 × 10^4^ cells cm^−2^ and astrocytes in the bottom chamber. During coculture, the TEER value was monitored every day with a Millicell ERS‐2 Epithelial Volt‐Ohm Meter (Millipore, USA). After 14 days, the chambers containing b. End3 cells were placed into new transwells to be cocultured with microglial cells on the bottom. Then, the BBB model could be used for further study.

##### Assessing the TNF‐*α* Suppression Effect by sCABs in the BBB Model

The b. End3 cells in transwell incubated with sCABs (65 ng Cy5‐ASO in 5 × 10^7^ particles) were analyzed at different time points: 0, 5, 15, and 30 min. The microglial cells in the bottom chamber were collected to quantify the ASO 24 h later. To assess the anti‐inflammatory effects, sCABs were added in to the apical chamber of transwells. LPS (500 ng *μ*L^−1^) was added to the bottom chamber 12 h post‐sCABs incubation. The microglial cell medium was collected 12 h later and centrifuged at 12 000 rpm for 30 min. The supernatant was collected to analyze the level of TNF‐*α* with an enzyme‐linked immune sorbent assay (ELISA) kit, and the morphology of microglial cells was observed with a Nikon confocal microscope (Nikon, Japan).

##### Transcytosis Analysis of sCABs

B16F10 cells were transfected with the lentiviral vector ( multiple of infection (MOI) = 5, catalog number LPP‐EGFP‐LV105, GeneCopoeia, USA) and screened with puromycin according to the manufacturer's instructions. The cells with high GFP expression were incubated with the cKGM/Cy5‐ASO complex and induced to undergo apoptosis as mentioned above. The sCABs containing both GFP and Cy5‐ASO were separated and added to the BBB model as described earlier. Then, the microglial cells in the bottom chamber were observed with a two‐photon laser confocal microscope (LSM 980 NLO with Airyscan2, Zeiss LSM980, Germany) 24 h after the incubation.

In BBB model, sCABs at dose of 5 × 10^7^ containing 65 ng Cy5‐ASO were incubated with TJs formed by b.End3 cells to analyze the transcytosis. The colocalization with transcytosis‐relevant proteins were analyzed at indicated time points. The endocytosis‐dependent proteins clathrin (anti‐clathrin heavy chain P1663 antibody, 2410S, CST, USA) and caveolin‐1 (anti‐caveolin‐1 antibody, 3238S, CST, USA) were examined at 5 min after the incubation. EEA‐1 (anti‐EEA‐1 antibody, BM4402, Boster, China), an intracellular marker of early endosomes, was tested at 10 min, as was Rab11 (anti‐Rab11B antibody, A04526‐1, Boster, China), a marker of recycling endosomes. Moreover, Snap23 (anti‐Snap23 antibody, BA2805, Boster, China), a membrane receptor involved in the interaction of endosomes with the basolateral membrane, was examined at 20 min.

##### Preparation of B16F10 Cells with CD44v6 Knockdown

The mouse CD44 full‐length DNA sequence was obtained from the website https://www.ncbi.nlm.nih.gov/genome/.The guide RNA (gRNA) for CD44v6 knockdown was designed by using the tools on the website http://crispr.mit.edu/ (donated by the Zhang Feng laboratory). The gRNA sequences are listed in Table S1, Supporting Information. The gRNA pairs were inserted into the lentiGuide plasmid (52963) with T_4_ DNA ligase.

The 293T cells were transfected with the plasmid lentiCas9‐Blast (plasmid 52962, 10 µg) or recombinant lentiGuide plasmid by Lipofectamine 2000 along with plasmids psPAX2 (plasmid 12260) and pMD2. G (plasmid 12259). Then, the lentivirus released from 293T cells were collected after 48 h culturation and concentrated with conventional methods. B16F10 cells were transfected with the concentrated lentivirus (MOI = 5) and screened for the high expression of clustered regularly interspaced short palindromin repeats associated (Case 9) nuclease with blasticidin and gRNA with puromycin. After screening, the B16F10 cells with CD44v6 knocked down by the CRISPR‐Cas 9 system were obtained. The knockdown efficiency was assayed by polymerase chain reactin (PCR) and western blot.

##### PCR Assay for CD44v6 Knockdown

Genomic DNA (gDNA) from both wild and engineered B16F10 cells was isolated with a gDNA extraction kit (DN1002, Aidlab, China) according to the manufacturer's instructions. The quantity and quality of DNA were measured by the NanoDrop One instrument (Thermo Scientific, USA). The DNA samples were amplified by PCR using AmpliTaq Gold Fast PCR Master Mix (Thermo Fisher Scientific, USA) on an Applied Biosystems ProFlex PCR system (Thermo Fisher Scientific, USA). The primers were designed according to the website https://www.ncbi.nlm.nih.gov/tools/primer‐blast/ , and the sequences are listed in Table S1, Supporting Information. The length of the predicted products was ≈522 bp. The PCR mixtures were then separated by 3% agarose gel electrophoresis and imaged with a UV transilluminator.

##### Western Blot Analysis

The protein samples were isolated with radio‐immunoprecipitation assay (RIPA) buffer containing 1% protease inhibitor (Roche, Switzerland), and the total concentrations were detected using a bicinchoninic acid (BCA) protein assay kit (MicroBCA Kit, Thermo Scientific, USA). The proteins mixed with SDS loading buffer were separated by sodium dodecyl sulfate‐polyacrylamide gel electrophoresis (SDS‐PAGE) and then transferred onto poly(vinylidine difluoride) membranes (Bio‐Rad, USA). The membranes were blocked with 5% skim milk and then incubated with primary antibodies (anti‐CD44v6, AB2080, Merck, Germany; anti‐*β*‐actin, BM0627, Boster, China) overnight at 4 °C. The membranes were incubated with HRP‐conjugated secondary antibodies (Life Technologies, USA) after three washes with PBST (PBS with 0. 1%v/v Tween‐20). After washing, the positive signals were visualized with fluorography using an enhanced chemiluminescence system (Cell Signaling Technology, USA).

##### Inflammatory PD Model Establishment and Treatment

Male C57BL/6J mice (8 weeks old) were anesthetized and fixed with a brain solid positioner (68025, RWD, China). LPS (*Escherichia coli* 0111:B4; Sigma, Germany) was stereotactically injected into the right side of the SNc for inflammatory PD model.^[^ ^40^ ^]^ Sterile PBS was injected into the left side of the SNc as a control. Mice 14 days after the injection of LPS showed symptoms of trembling, listless with erect hair, indicating the success of PD model preliminarily.

To evaluate the preventive effects of sCABs, male C57BL/6 mice were intravenously injected with sCABs (2.7 × 10^9^ particles containing 3.5 µg ASO) for four times every three days, and another six injections were carried out after the stereotaxic injection of LPS. To assess the therapeutic effects of sCABs, the treatment of inflammatory PD mice was started on the day after the stereotaxic injection of LPS. The sCABs at dose of 2.7 × 10^9^ particles containing 3.5 µg ASO were separately injected into tail vein of PD mice every other day for ten times. Mice injected with PBS were chosen as the control. After the last injection of sCABs, mice were measured for behavioral test. Mice were sacrificed at day 3 after the last injection and the brain tissue was harvested for immunohistopathological examination, RT‐qPCR and cytokines analysis (TNF‐*α*, IFN‐*γ*, IL‐1*β*, and IL‐6) using ELISA kits according to the manufacturer's instructions (eBioscience, USA). Meanwhile, the primers for TNF‐*α* and *β*‐action are listed in Table S1, Supporting Information.

##### Behavioral Tests

After the last injection of sCABs, mice were measured for behavioral test.^[^ ^41^ ^]^ In rotarod test, mice were trained during the adaptive phase before LPS injection and the speed of instrument was set at 30 rpm min^−1^. Then, mice with normal movement time (>7 min) were chosen for PD model and measured for movement time after different treatments. For vertical episodes, mice were measured in the dark and quiet room after 30 min adapting. A vertical episode was recorded when both of forelegs was off the ground. The climbing test was conducted with a ball‐and‐stick equipment. The time of mice climbing from the ball to the bottom of pole was measured. For behavioral test, all mice were tested for three independent times. The behavioral tests were performed during the light cycle (10:00 to 17:00). Before tested, mice were put in the testing room for sixty‐minute adapting.

##### H&E Staining

Brains and other five organs (heart, liver, spleen, kidney, and lung) were excised from mice six months after the treatment with 2.7 × 10^9^ sCABs containing 3.5 µg for 10 times. All tissues were sliced at 5 µm thickness after paraffin embedding and stained with H&E according to the manufacturer's instructions (Jiancheng Bioengineering Institute, China). The images were captured with an Eclipse Ni‐E upright microscope (Nikon, Japan).

##### Statistical Analysis

All data were normally distributed. Data are shown as mean ± SD. The sample size (*n*) was set up according to the pre‐experimental data and *n* ≥ 3 per group. Student's *t* test and one‐way analysis of variance (ANOVA) followed by Bonferroni's multiple comparison test were performed. A *p* value < 0.05 was considered significant. The results were statistically analyzed using Prism software (GraphPad 8.0.2, USA).

## Conflict of Interest

The authors declare no conflict of interest.

## Author Contributions

Y.L.W., J.Y.P., Q.Y.W., and L.C.Y. performed the experiments. L.T.W. and Z.X. assisted in designing this study. C.M.W. assisted in designing this study and writing the manuscript. L.D. and J.F.Z. designed this study and prepared the manuscript.

## Supporting information

Supporting Information

Supporting Video 1

Supporting Video 2

Supporting Video 3

Supporting Table 1

Supporting Table 2

Supporting Table 3

## Data Availability

The data that support the findings of this study are available in the Supporting Information of this article or from the corresponding author upon reasonable request.
