# Supplementary material for: Delivering Antisense Oligonucleotides across the Blood‐Brain Barrier by Tumor Cell‐Derived Small Apoptotic Bodies
Source: Adv Sci (Weinh). 2021 May 4;8(13):2004929. doi: 10.1002/advs.202004929 (PMC8261483; doi:10.1002/advs.202004929)
Supplement: Supplementary file 1 — Supporting Information [file ADVS-8-2004929-s001.pdf]

© 2021 Wiley-VCH GmbH

ADVANCED SCIENCE
Open Access

Supporting Information

for *Adv. Sci.*, DOI: 10.1002/advs.202004929

Delivering Antisense Oligonucleotides across the Blood-Brain Barrier by Tumor Cell-Derived Small Apoptotic Bodies

Yulian Wang, Jiayun Pang, Qingyun Wang, Luocheng Yan, Lintao Wang, Zhen Xing, Chunming Wang, Junfeng Zhang*, Lei Dong**

Supporting Information

Delivering Antisense Oligonucleotides across the Blood-Brain Barrier by Tumor Cell-Derived Small Apoptotic Bodies

Yulian Wang, *Jiayun Pang*, *Qingyun Wang, Luocheng Yan*, *Lintao Wang*, *Zhen Xing*, *Chunming Wang*[*], *Junfeng Zhang*[*], *Lei Dong*[*]

Yulian Wang, Jiayun Pang, Luocheng Yan, Lintao Wang, Zhen Xing, Prof. Junfeng Zhang, Prof. Lei Dong.
State Key Laboratory of Pharmaceutical Biotechnology, School of Life Sciences, Nanjing University, 163 Xianlin Avenue, Nanjing 210093, China.

Prof. Chunming Wang
State Key Laboratory of Quality Research in Chinese Medicine, Institute of Chinese Medical Sciences, University of Macau, Taipa, Macau SAR, China.

Correspondence: Email: CMWang@um.edu.mo; Email: jfzhang@nju.edu.cn;
Email: leidong@nju.edu.cn.

Supporting Information

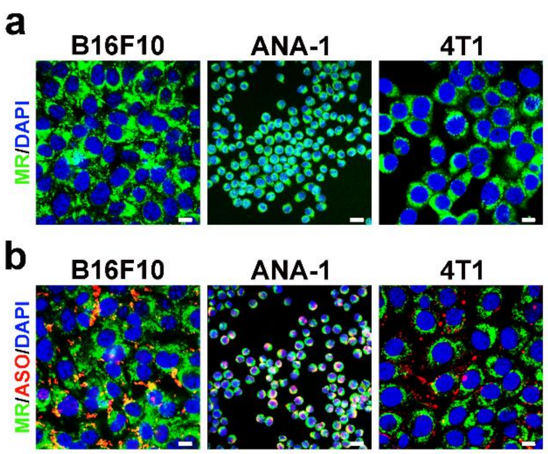

Figure S1. Immunofluorescence detection and cell transfection assay. a. Representative fluorescence images of MR expression in the three cell lines. Scale bar: 25 μm. b. Representative fluorescence microscopy images of three cell lines transfected with CKAs. Scale bar: 25 μm. Images are representative of three independent experiments.

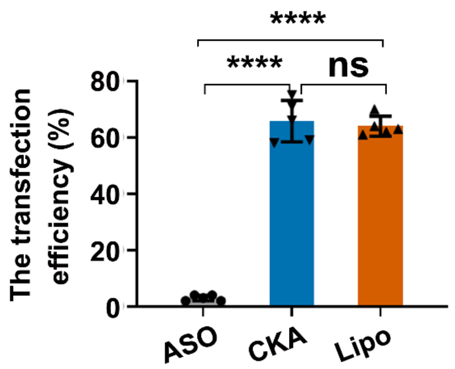

Figure S2. The cell transfection efficiency assay. B16F10 cells were incubated with naked ASO, the complex of cKGM/ASO or the complex of Lipofectamine 2000 /ASO and 6 h later the cells were collected for RT-qPCR analysis. Representative results are presented as the means ± SD; n = 5 per group. P-values are calculated using one-way ANOVA followed by Bonferroni's multiple comparisons post hoc test, ****$P < 0.0001$.

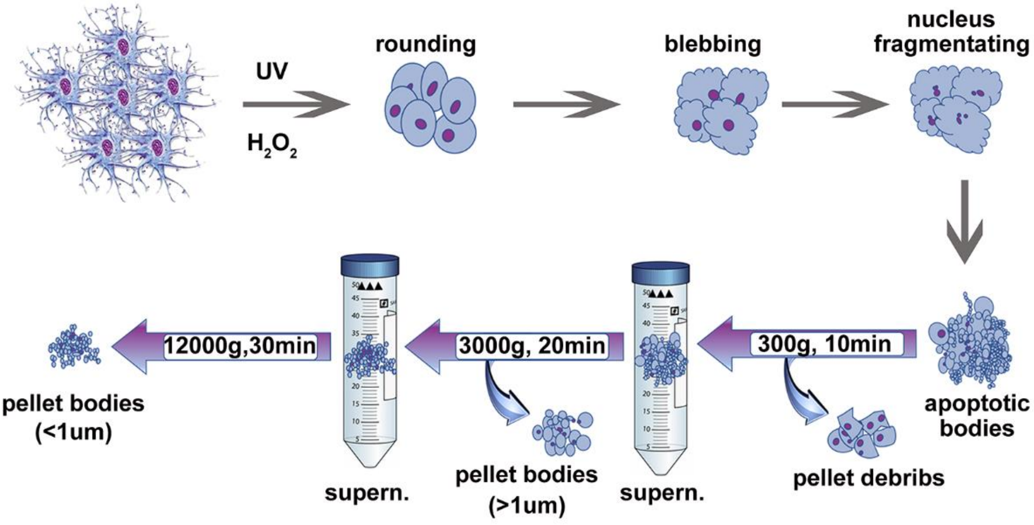

Figure S3. The gradient separation process for sCABs.

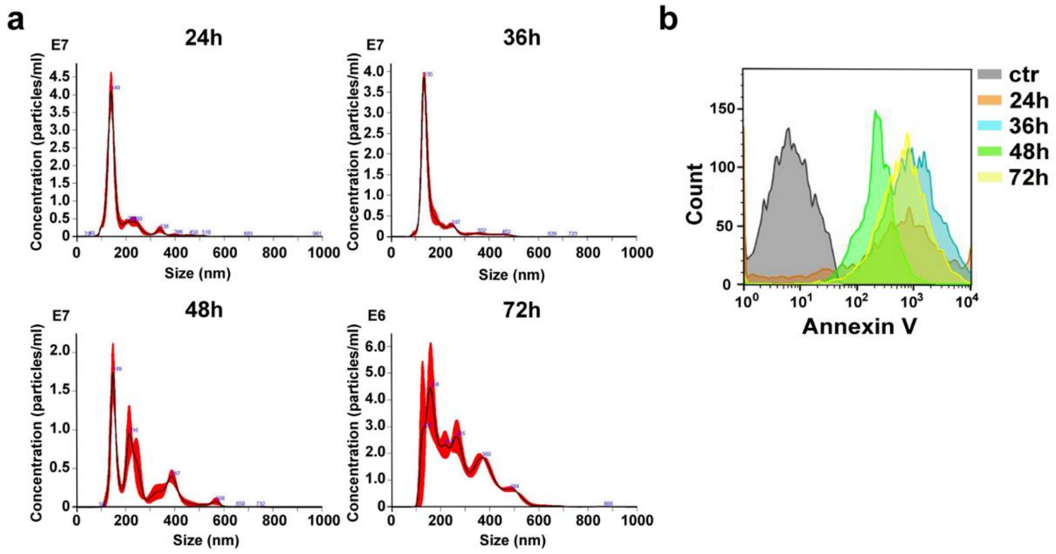

Figure S4. Characterization of sCABs from B16F10 cells collected at different times. a. The diameter distributions of sCABs. b. The Annexin V^+ ratios of sCABs.

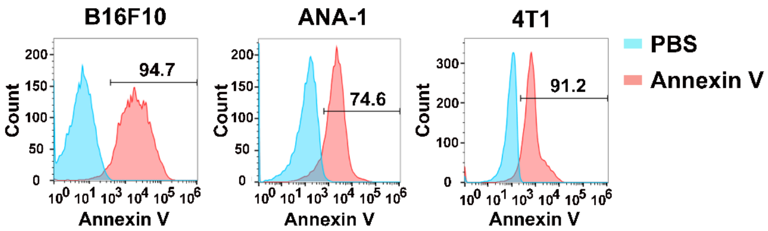

Figure S5. The Annexin V^+ ratios of sCABs collected from three cell lines at 36 h after apoptosis induction.

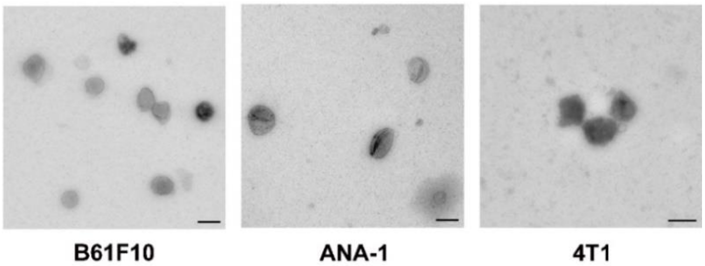

Figure S6. The TEM images of sCABs in three cell lines collected at 36h after apoptosis induction. The sCABs stained with uranyl-oxalate solution and then with methyl cellulose-UA showed different degrees of coloration owing to the inhomogeneous distribution of biomolecules, including nucleic acids, proteins and lipids. Scale bars: 200 nm. Images are representative of three independent experiments.

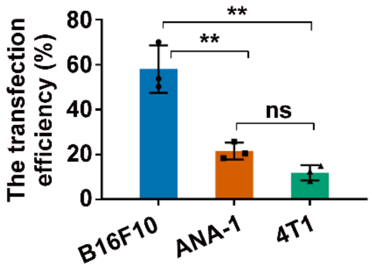

Figure S7. The transfection efficiencies of three cells for ASO. Representative results are presented as the means ± SD; n = 3 per group. P-values are calculated using one-way ANOVA followed by Bonferroni's multiple comparisons post hoc test, $^{**}P < 0.01$.

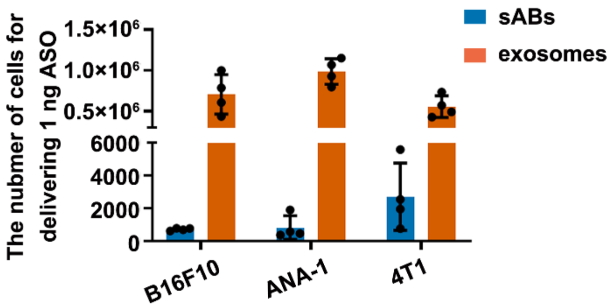

Figure S8. The numbers of cells used for collecting sCABs and exosomes containing 1 ng ASO. Representative results are presented as the means ± SD; n = 4 per group.

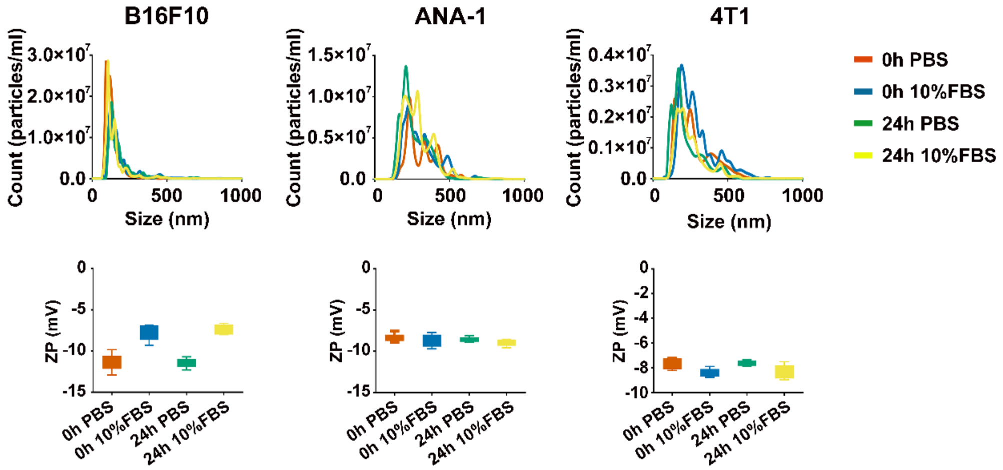

Figure S9. The stability tests of sCABs. a. The diameter distributions of sCABs in pure PBS or PBS supplemented with 10% FBS after 24 h. b. The ZP values of sCABs.

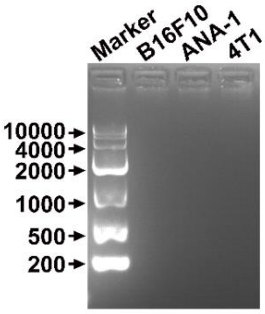

Figure S10. Images of DNA in sCABs from three cell lines using a Gel Imager System.

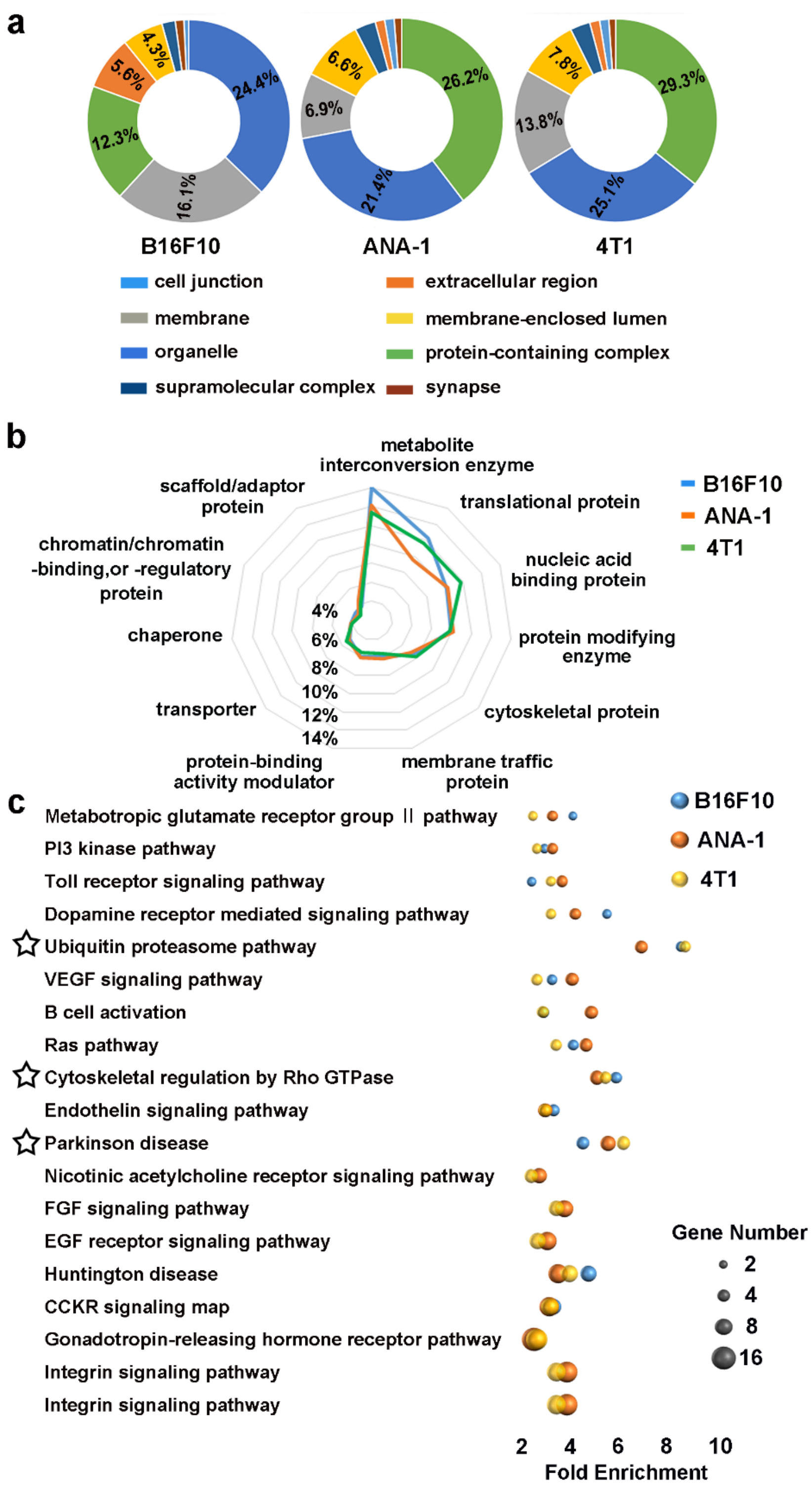

Figure S11. The classification of proteins from sCABs identified by MS**.** a. The classification of proteins according to cellular components. b. The classification of proteins according to function. c. KEGG pathway analysis of the gene number (≥ 2) and fold enrichment (≥ 2) for proteins in three sCABs and the pathways highly concentrated in three sCABs were marked with stars.

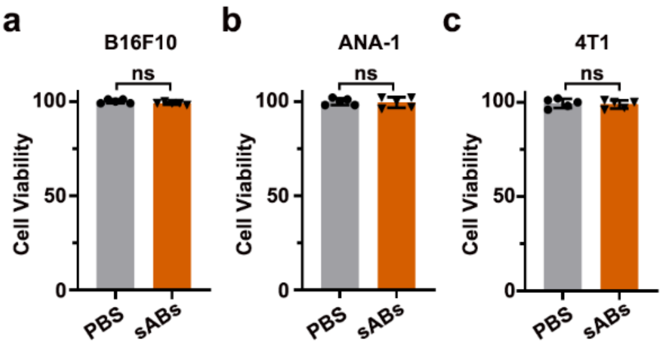

Figure S12. Cell viability of 1 × 10^4 b.End3 cells after incubation with 1 × 10^6 sCABs derived from three cell lines for 12 h. Representative results are presented as the means ± SD; n = 5 per group. *P* value was measured using Student's *t*-test, **P* < 0.05.

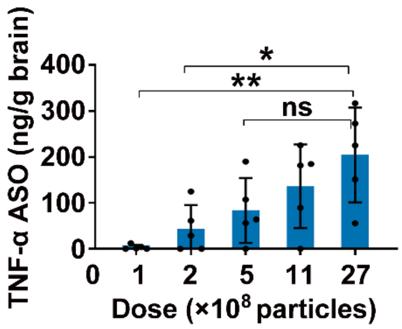

Figure S13. The dose-dependent analysis of ASO in the brain from mice treated with different amounts of sCABs 24 h post administration. Representative results are presented as the means ± SD; n = 5 per group. *P* value as measured using one-way ANOVA followed by Bonferroni's multiple comparisons post hoc test, **P* < 0.05, ***P* < 0.01.

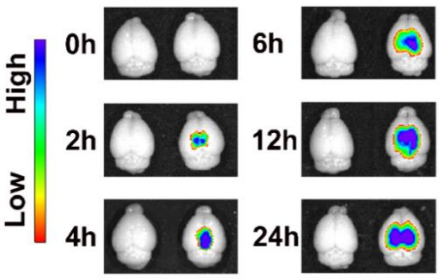

Figure S14. Examination of brains harvested from mice that received sCABs loaded with Cy5-ASO by the IVIS Lumina XR small animal imaging system. The dose of ASO was 3.5 μg delivered by 2.7 × 10^9 sCABs. The excitation and emission wavelengths were 640 nm and 670 nm, respectively.

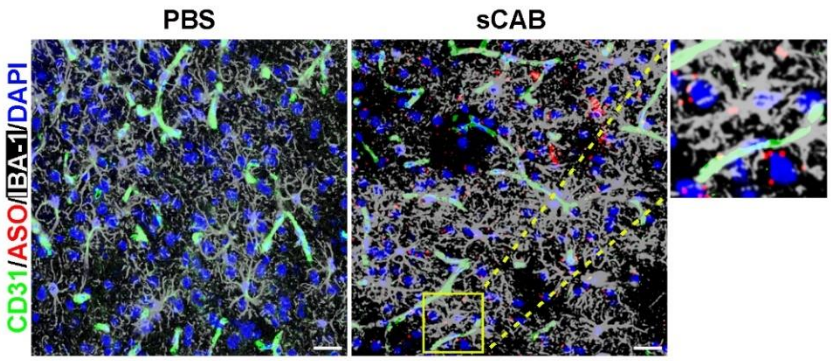

Figure S15. Representative fluorescence microscopy images of brain tissue from mice 20 min after the injection of 2.7 × 10^9 sCABs containing 3.5 μg ASO. The colocalization of Cy5-ASO with microglial cells and vessels were shown. The right panel shows a magnification of the area selected by the yellow square. Scale bar: 25 μm. Images are representative for three independent experiments.

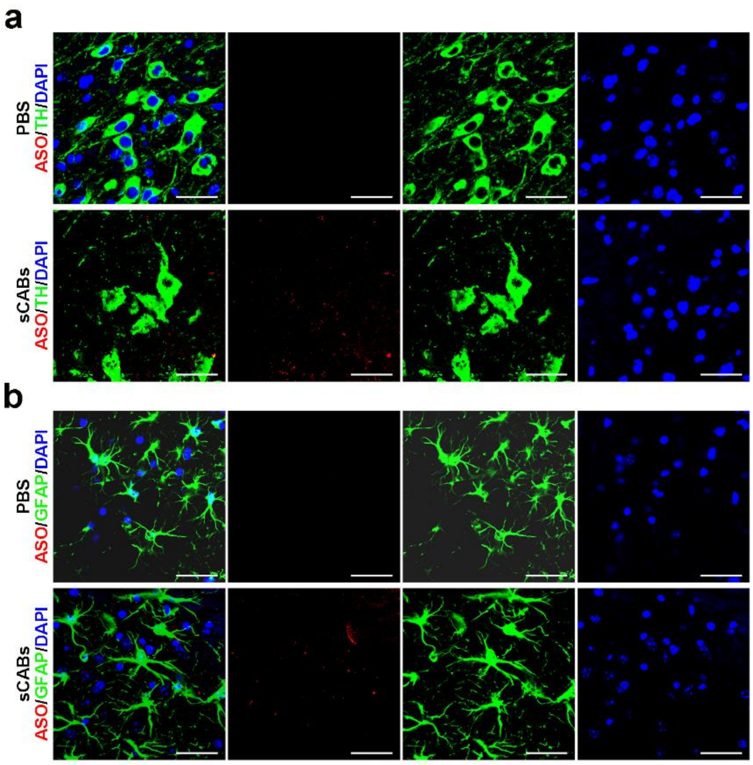

Figure S16. Representative fluorescence microscopy images of brain tissue from mice 20 min after the injection of 2.7 × 10^9 sCABs containing 3.5 μg ASO. The colocalization of Cy5-ASO with TH^+ neurons (a) and astrocytes (b) were shown. Scale bar: 25 μm. Images are representative for three independent experiments.

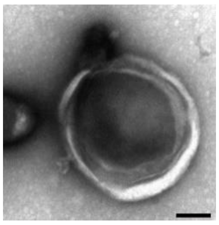

Figure S17. TEM image of a piece of a sCAB with an intact membrane structure in vitro. Scale bar: 100 nm. Images are representative for three independent experiments.

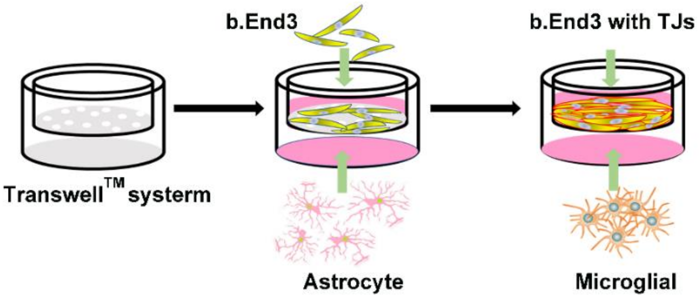

Figure S18. Schematic drawing of the two-week organization of b. End3 cells, astrocytes and microglial cells in the BBB triculture model.

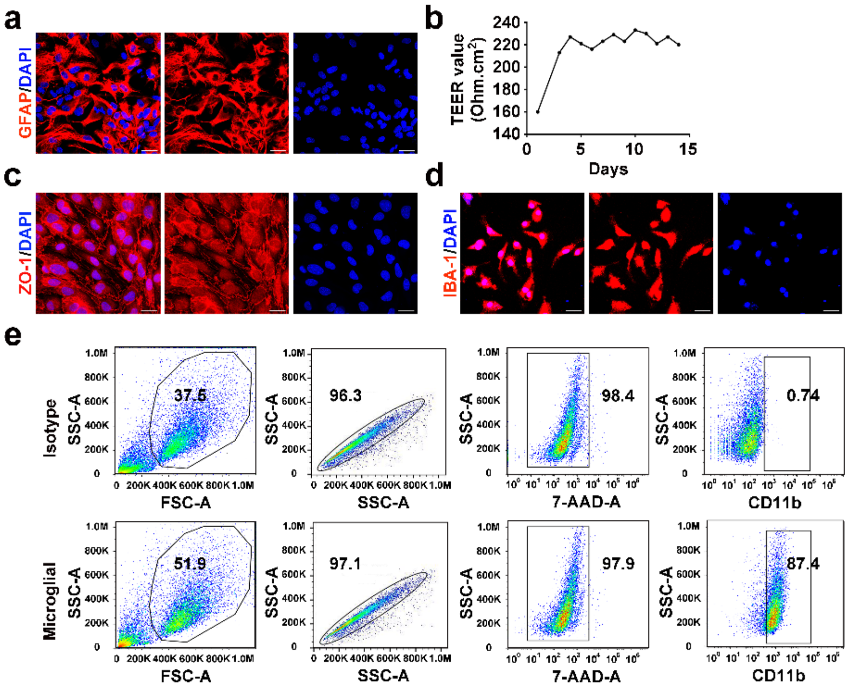

Figure S19. The characterization of cells in the BBB model. a. Representative fluorescence microscopy images of primary astrocytes. Scale bar: 25 μm. b. The TEER values of b. End3 cells cocultured with primary astrocytes for 14 days in the BBB model. c. Representative fluorescence microscopy images showing the TJs in b. End3 cells after fourteen days of coculture. Scale bar: 25 μm. d. Representative fluorescence

microscopy images of microglial cells. Scale bar: 25 μm. e. Gating strategy to identify CD11b-positive microglial cells. Cells were gated as CD11b^{+} live single cells. Isotype: isotype control. Images are representative for three independent experiments.

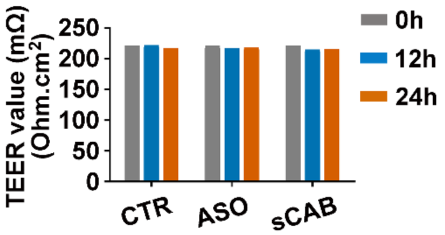

Figure S20. The TEER values of b. End3 cells in the BBB model treated with naked and sCABs-delivered ASO after 12 h and 24 h.

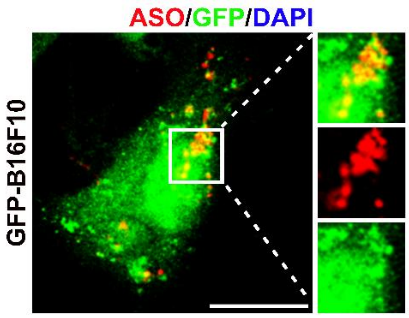

Figure S21. Representative fluorescence microscopy images showing the expression of GFP in sCABs along with Cy5-ASO. The right panels show magnifications of the area selected by the white square. Scale bar: 25 μm. Images are representative for three independent experiments.

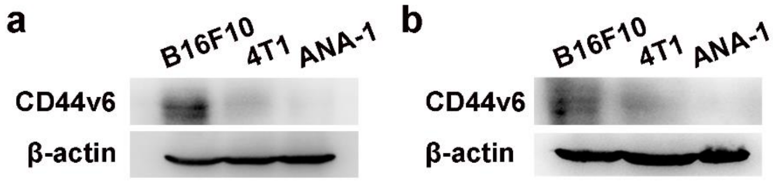

Figure S22. The expression of CD44v6 protein in three cell lines and their sCABs. a. Western blot showing the CD44v6 protein expression in the three cell lines. b. Western blot showing the CD44v6 protein in sCABs from three cell lines.

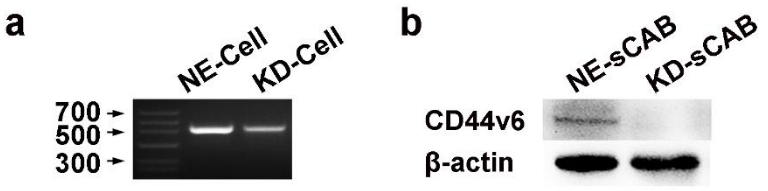

Figure S23. CD44v6 was knocked down in B16F10 cells. a. Image showing the reduced expression of CD44v6 in DNA samples from B16F10 cells as determined using a Gel Imager System. b. Western blot showing reduced expression of CD44v6 in B16F10 cells. NE-Cell: B16F10 cells with normal expression of CD44v6. KD-Cell: B16F10 cells with CD44v6 knockdown. NE-sCAB: sCABs from NE cells, KD-sCAB: sCABs from KD cells.

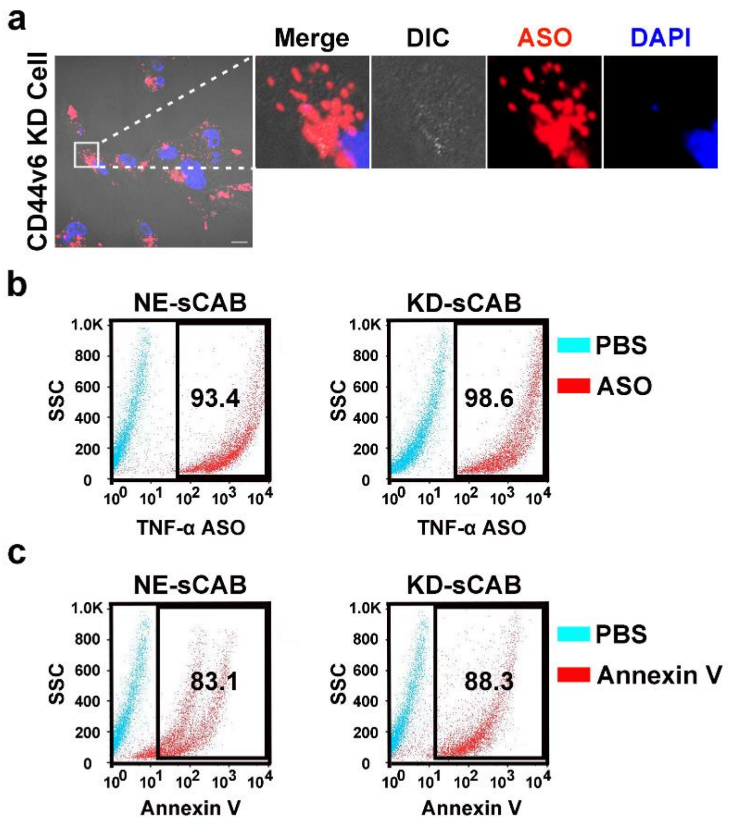

Figure S24. Characterization of sCABs from cells with CD44v6 knockdown. a. Representative fluorescence microscopy images of sCABs from B16F10 cells with CD44v6 knockdown. Scale bar: 25 μm. b. The TNF-α ASO positive ratios of normal and CD44v6-knockdown sCABs. c. The ratios of Annexin V^+ for normal and CD44v6-knockdown sCABs. Images are representative for three independent experiments.

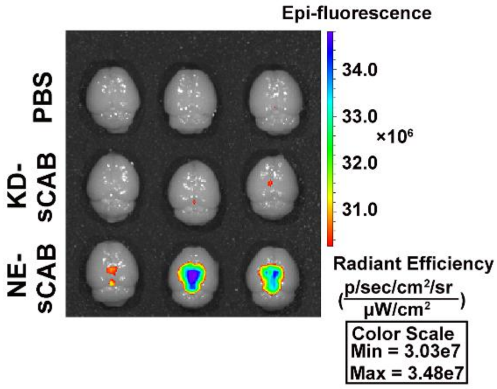

Figure S25. Examination of brains harvested from mice receiving normal and CD44v6-knockdown sCABs loaded with Cy5-ASO by the IVIS Lumina XR small animal imaging system. The brain tissues were excised 24 h after an injection of 2.7×10^9 sCABs containing 3.5 μg ASO. The excitation and emission wavelengths were 640 nm and 670 nm, respectively

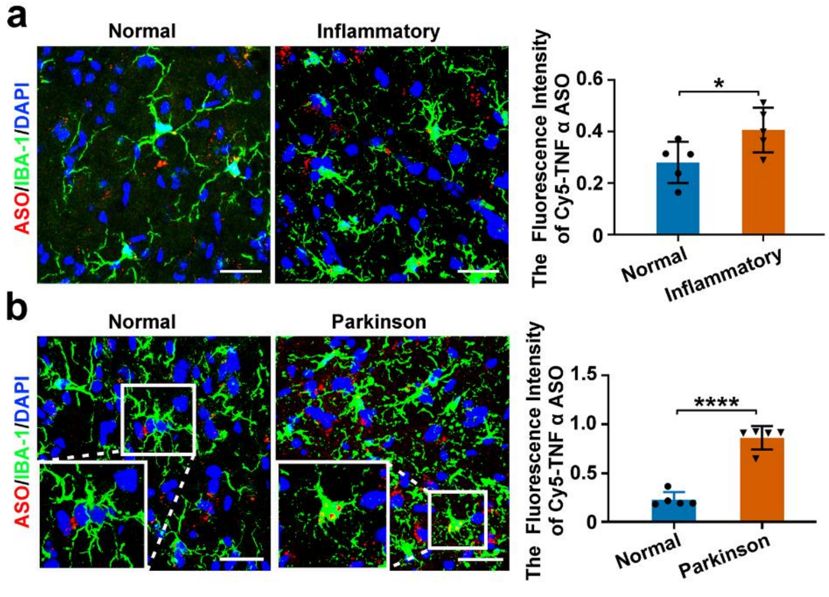

Figure S26. Representative fluorescence microscopy images of increased ASO in microglial cells along with brain inflammation. a. The brain showed mild inflammatory reactions on the 7th day after LPS injection. b. The brain developed Parkinson's disease and showed worse inflammation on the 14th day after LPS injection. The panels on the lower left show magnifications of the areas selected by the white squares. Scale bar: 25 μm. The fluorescence intensity of Cy5-ASO in brain sections were measured with Image J and analyzed. Images are representative for three independent experiments. Representative results are presented as the means ± SD; n = 5 per group. *P* value was

measured using Student's t-test, $*P < 0.05$, $****P < 0.0001$.

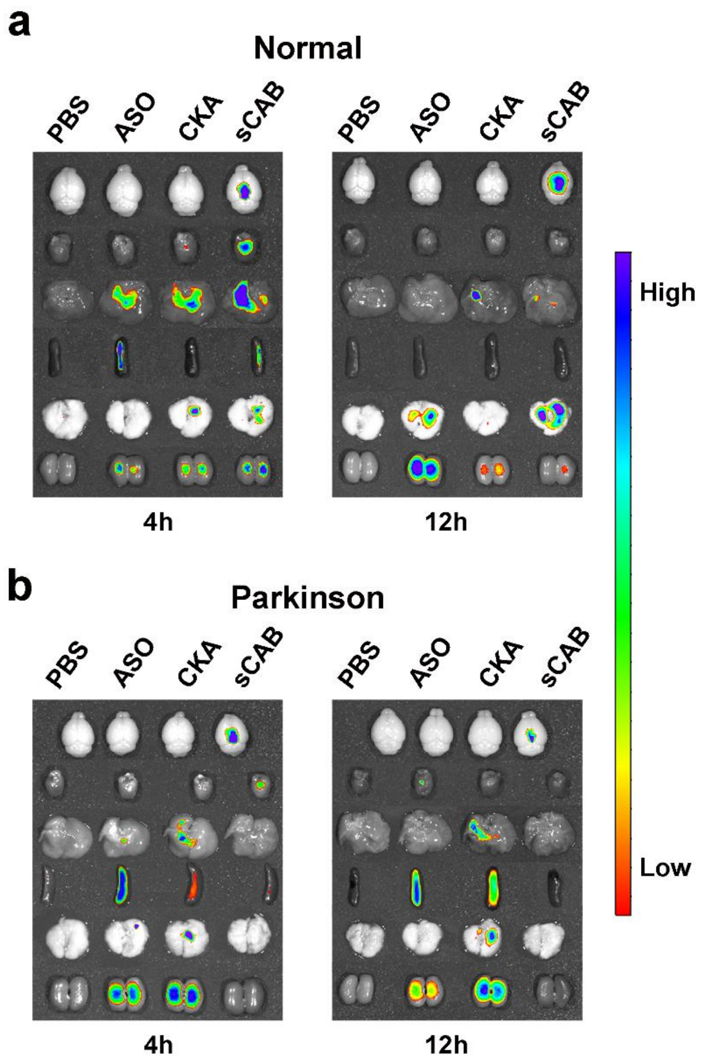

Figure S27. Examination of organs harvested from normal and PD model mice receiving sCABs loaded with Cy5-ASO by the IVIS Lumina XR small animal imaging system. ASO: naked ASO, CKA: cKGM/ASO complex, sCAB: sCABs loaded with CAK. From top to bottom, the organs are arranged in the following order: brain, heart, liver, spleen, lung and kidney. The excitation and emission wavelengths were 640 nm and 670 nm, respectively

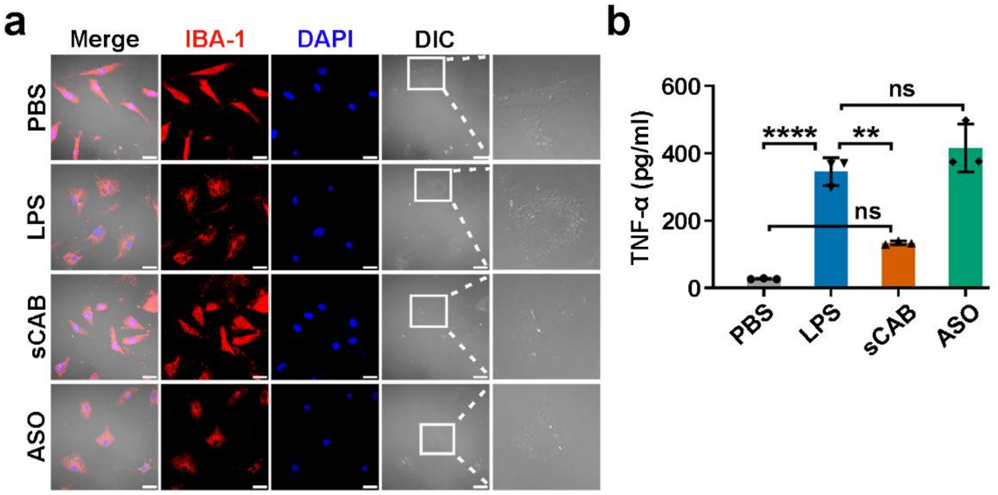

Figure S28. The anti-inflammatory effect of sCABs (65 ng ASO delivered by 5×10^7 sCABs) *in an in vitro* BBB model. a. Representative fluorescence microscopy images showing the morphologies of microglial cells under different treatments 24 h after sCABs-incubation. The panels on the right show magnifications of the areas selected by the white squares. Scale bar: 25 µm. Images are representative for three independent experiments. b. ELISA of TNF-α in microglial cells. LPS: LPS + PBS, sCAB: LPS + sCABs, ASO: LPS + naked ASO. Representative results are presented as the means ± SD; n = 3 per group. *P* value was measured using one-way ANOVA followed by Bonferroni's multiple comparisons post hoc test, *$P < 0.05$, **$P < 0.01$, ***$P < 0.001$, ****$P < 0.0001$.

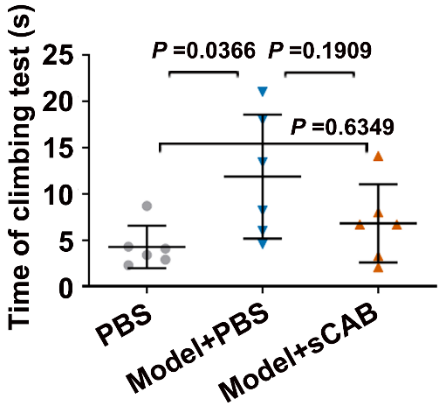

Figure S29. The climbing test for mice with different treatments. Representative results are presented as the means ± SD; n = 6 per group. *P* value was measured using one-way ANOVA followed by Bonferroni's multiple comparisons post hoc test.

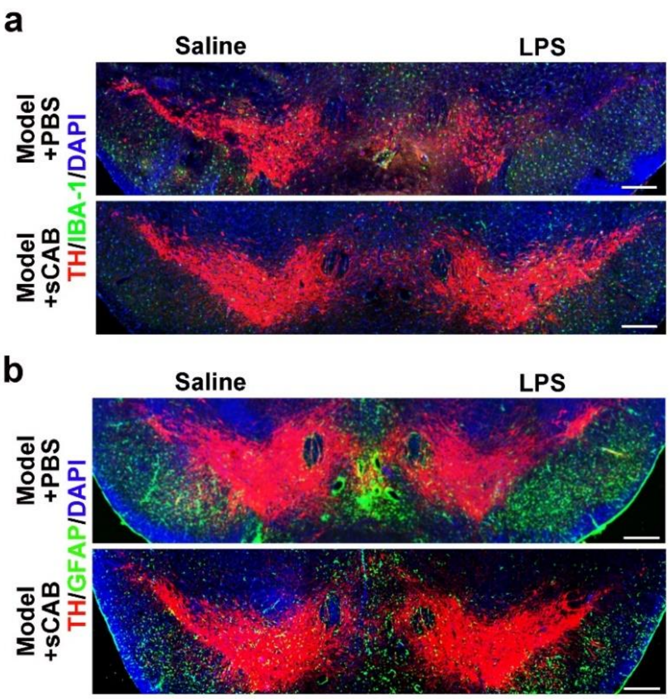

Figure S30. Representative fluorescence microscopy images showing the morphological changes in microglial cells (a) and astrocytes (b) in the SNc area. Scale bar: 250 μm. Images are representative for three independent experiments.

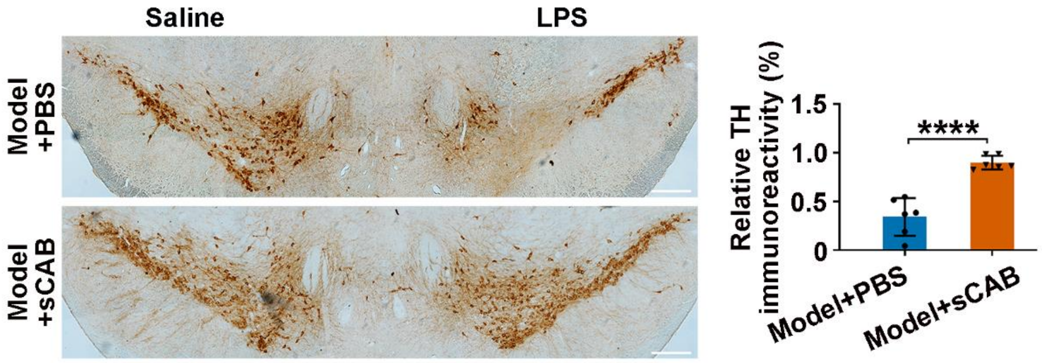

Figure S31. Representative microscopy images showing the improvement of TH^+ DA neurons in the SNc area after sCABs treatment and statistical analysis with ImageJ. Scale bar: 250 μm. Images are representative for three independent experiments. Representative results are presented as the means ± SD; n = 6 per group. *P* value was measured using Student's *t*-test, ****$P < 0.0001$.

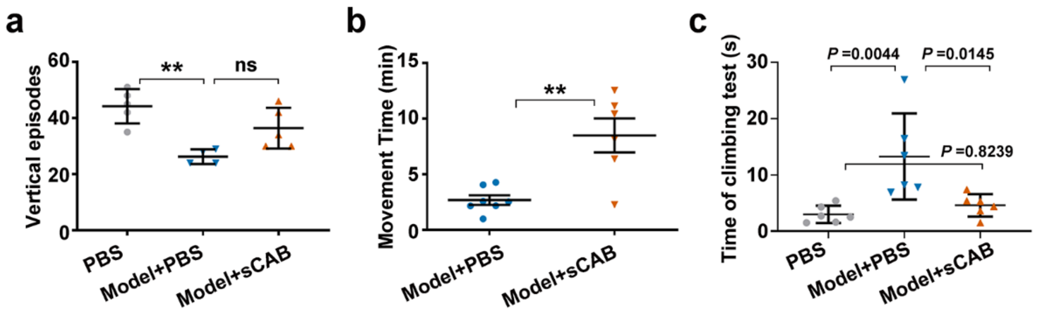

Figure S32. Vertical episodes (a), movement times (b) and climbing test of mice (c). Representative results are presented as the means ± SD; n ≥ 5 per group. *P* value was as measured using one-way ANOVA followed by Bonferroni's multiple comparisons post hoc test (a, c) and Student's *t*-test (b), **P* < 0.05, ***P* < 0.01.

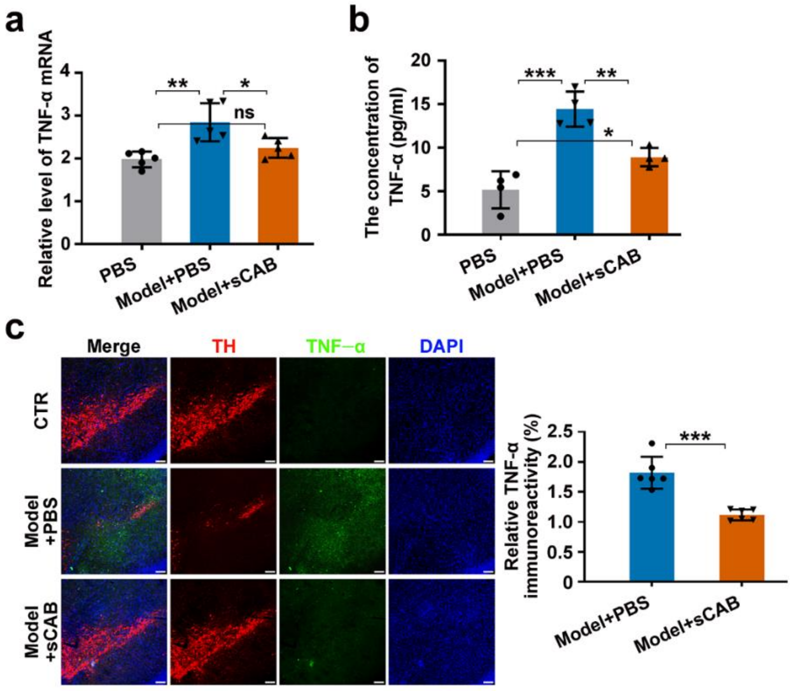

Figure S33. Expression of TNF-α in the brains of mice administered different treatments. a-b. The TNF-α mRNA (a) and protein (b) levels in the mouse brain. c. Representative fluorescence images showing the expression of TNF-α in the brains of mice that received different treatments and statistical analysis of the fluorescence intensity. Scale bar: 100 μm. Images are representative for three independent experiments. Representative results are presented as the means ± SD; n ≥ 4 per group. *P* value was measured using ANOVA followed by Bonferroni's multiple comparisons post hoc test (a, b) and Student's *t*-test (c), **P* < 0.05, ***P* < 0.01, ****P* < 0.001.

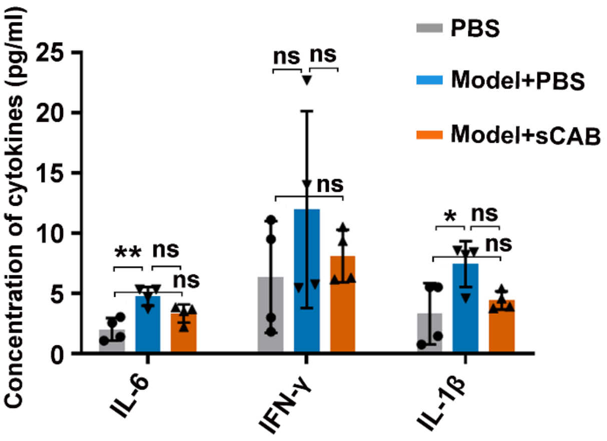

Figure S34. Quantification of the 3 proinflammatory cytokines in the mouse brains after the treatments. Representative results are presented as the means ± SD; n = 4 per group. *P* value was measured using one-way ANOVA followed by Bonferroni's multiple comparisons post hoc test, **P* < 0.05, ***P* < 0.01.

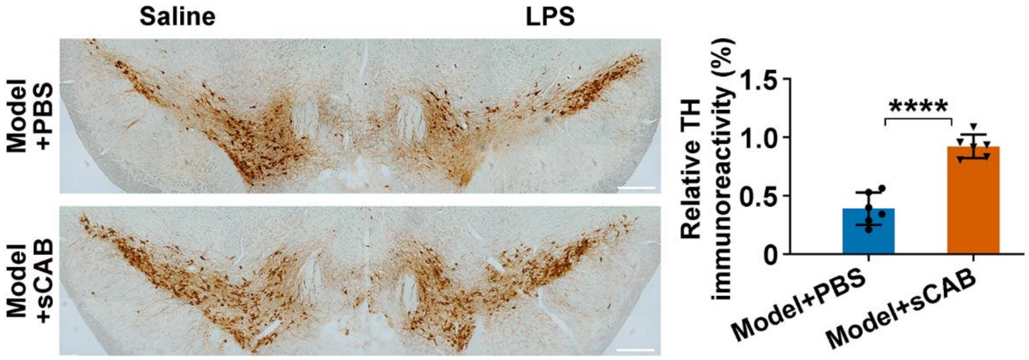

Figure S35. Representative microscopy images showing the improvement of TH^+ DA neurons in the SNc area with different treatments and statistical analysis with ImageJ software. Scale bar: 250 μm. Images are representative for three independent experiments. Representative results are presented as the means ± SD; n = 6 per group. *P* value was measured by Student's *t*-test, *****P* < 0.0001.

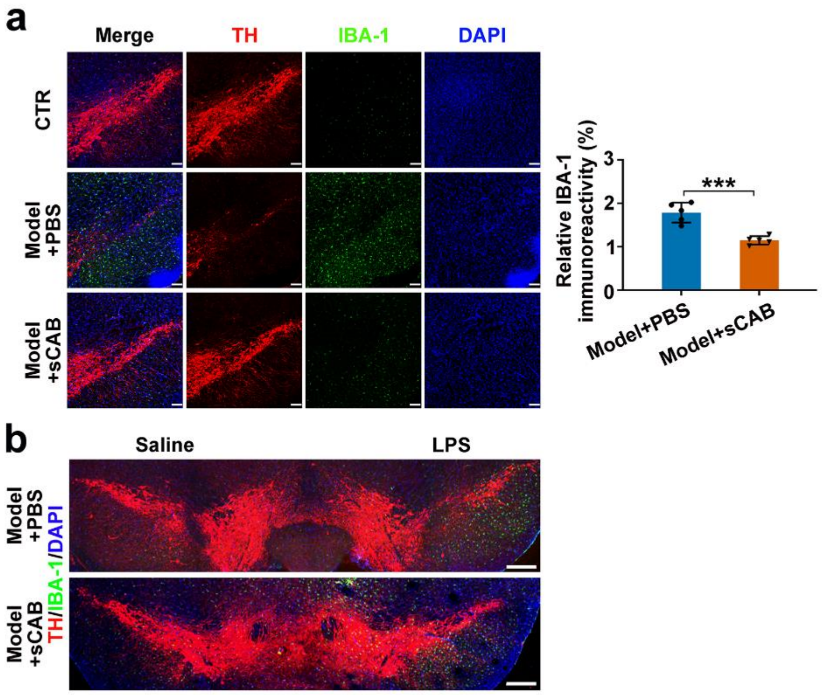

Figure S36. The morphological changes of microglial cells after different treatment. a. Representative fluorescence images showing the morphological changes in microglial cells and statistical analysis of the fluorescence intensity. Scale bar: 100 μm. b. Representative fluorescence images showing the morphological changes in microglial cells in the whole SNc area. Scale bar: 250 μm. Images are representative for three independent experiments. Representative results are presented as the means ± SD; n = 5 per group. *P* value was measured by Student's *t*-test, ***$P < 0.001$.

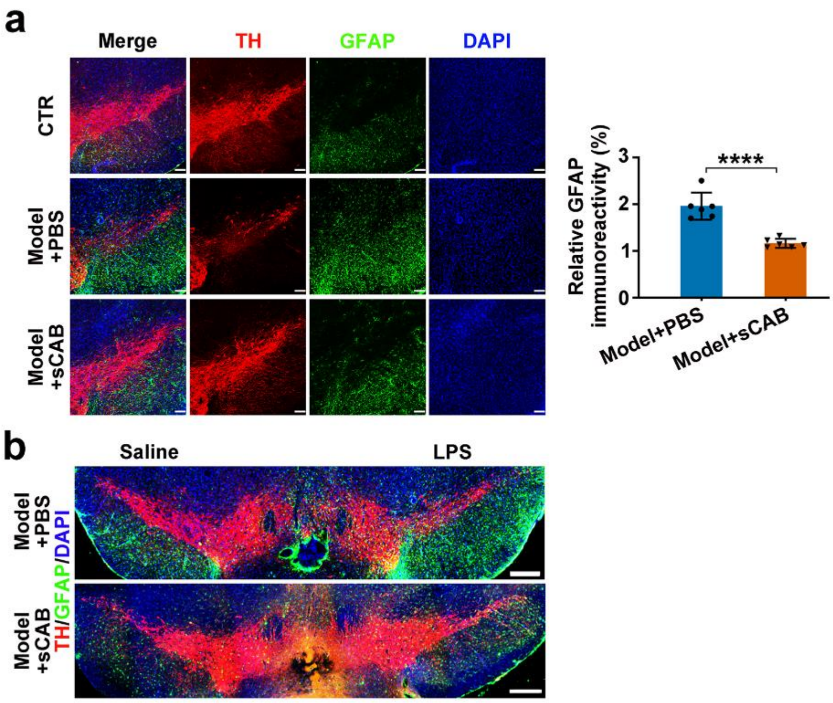

Figure S37. The morphological changes of astrocytes after different treatment. a. Representative fluorescence images showing the morphological changes in astrocytes and statistical analysis of the fluorescence intensity. Scale bar: 100 μm. b. Representative fluorescence images showing the morphological changes in astrocytes in the whole SNc area. Scale bar: 250 μm. Images are representative for three independent experiments. Representative results are presented as the means ± SD; n = 6 per group. *P* value was measured by Student's *t*-test, ****$P < 0.0001$.

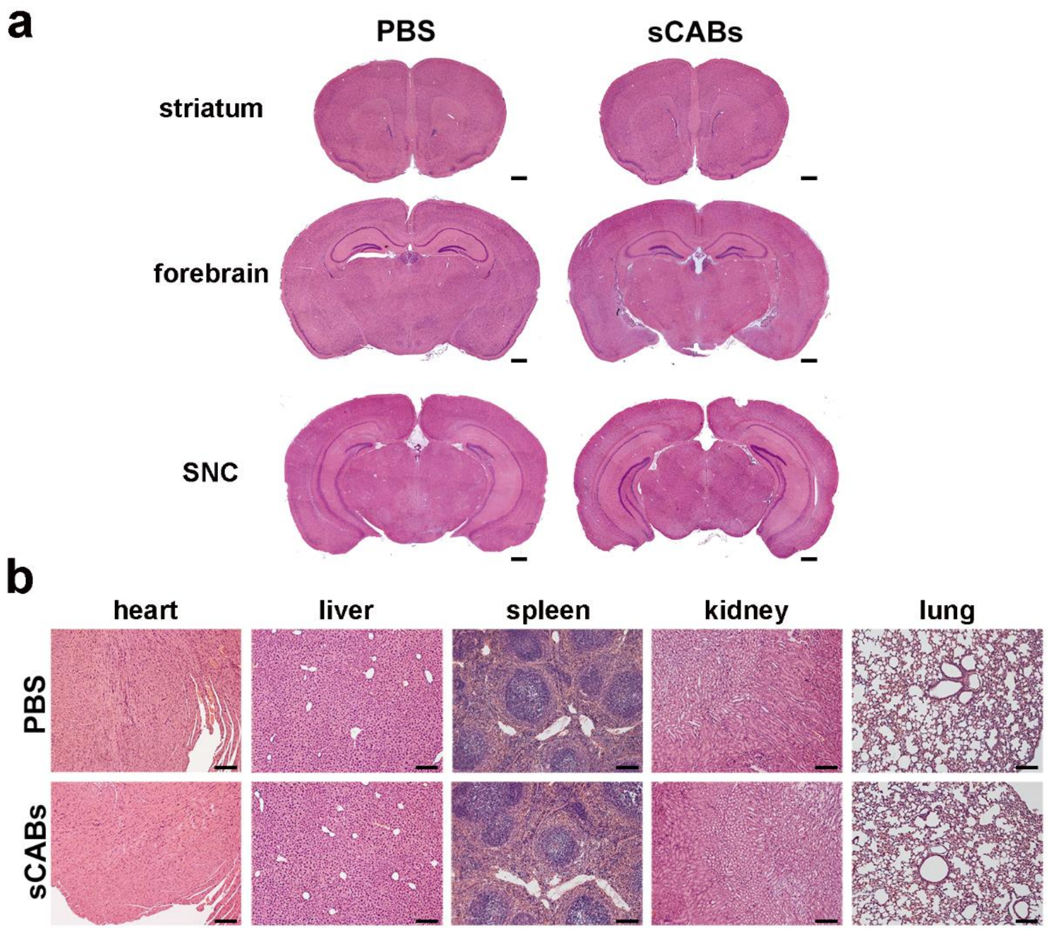

Figure S38. The histopathological examination of different tissues after sCABs treatment. Three parts of brain (a) and the main five peripheral organs (b) from mice treated with sCABs (2.7×10^9 particles containing 3.5 μg ASO for ten times) were analyzed with H&E staining six months later, Scale bar: 500 μm (a) and 100 μm (b). Images are representative for three independent experiments.

Supporting Information

Table 1. The Primer Sequences applied in the work.

	Forward (5'-3')	Reverse (5'-3')
RT primer for ASO	GTCTGTATGCTTGTTCTCGTCTCTGTGTCATCCCTCAAGCATACA GACGTGGTG	
Primer set for ASO	TTACATATAACCCATCGGCTGG	TATGCTTGTTCTCGTCTCTGTGTC
g-RNA	CACCGAGACGTGGTTTCAGAACGGA	AAACTCCGTTCTGAAACCACGTCTC
CD44 exon 11	ATTCTGTATCTATCATCTATGTATGTTTATT	TCCTTTGTGTGCTTCTATCTAT
TNF-α	CGTGGAACTGGCAGAAGAGG	GGAATGAGAAGAGGCTGAGACA
β–actin	GACCTCTATGCCAACACAGTGC	GTACTCCTGCTTGCTGATCCAC
